# Development of a cobalt(iii)-based ponatinib prodrug system†

**DOI:** 10.1039/d1qi00211b

**Published:** 2021-03-30

**Authors:** Marlene Mathuber, Michael Gutmann, Mery La Franca, Petra Vician, Anna Laemmerer, Patrick Moser, Bernhard K. Keppler, Walter Berger, Christian R. Kowol

**Affiliations:** Institute of Inorganic Chemistry, Faculty of Chemistry, University of Vienna Waehringer Straße 42 1090 Vienna Austria; Institute of Cancer Research and Comprehensive Cancer Center, Medical University of Vienna Borschkegasse 8A 1090 Vienna Austria; Department of Biological, Chemical and Pharmaceutical Sciences and Technologies, University of Palermo via Archirafi 32 90123 Palermo Italy; Research Cluster “Translational Cancer Therapy Research”, University of Vienna and Medical University of Vienna 1090 Vienna Austria christian.kowol@univie.ac.at walter.berger@meduniwien.ac.at

## Abstract

Receptor tyrosine kinase inhibitors have become a central part of modern targeted cancer therapy. However, their curative potential is distinctly limited by both rapid resistance development and severe adverse effects. Consequently, tumor-specific drug activation based on prodrug designs, exploiting tumor-specific properties such as hypoxic oxygen conditions, is a feasible strategy to widen the therapeutic window. After proof-of-principal molecular docking studies, we have synthesized two cobalt(iii) complexes using a derivative of the clinically approved Abelson (ABL) kinase and fibroblast growth factor receptor (FGFR) inhibitor ponatinib. Acetylacetone (acac) or methylacetylacetone (Meacac) have been used as ancillary ligands to modulate the reduction potential. The ponatinib derivative, characterized by an ethylenediamine moiety instead of the piperazine ring, exhibited comparable cell-free target kinase inhibition potency. Hypoxia-dependent release of the ligand from the cobalt(iii) complexes was proven by changed fluorescence properties, enhanced downstream signaling inhibition and increased *in vitro* anticancer activity in BCR-ABL- and FGFR-driven cancer models. Respective tumor-inhibiting *in vivo* effects in the BCR-ABL-driven K-562 leukemia model were restricted to the cobalt(iii) complex with the higher reduction potential and confirmed in a FGFR-driven urothelial carcinoma xenograft model. Summarizing, we here present for the first time hypoxia-activatable prodrugs of the clinically approved tyrosine kinase inhibitor ponatinib and a correlation of the *in vivo* activity with their reduction potential.

## Abstract 2

This study presents the first hypoxia-activatable cobalt(iii) prodrugs of the clinically approved tyrosine kinase inhibitor ponatinib. The chemical properties of the complexes have been correlated with the *in vivo* antitumor activity.

## Graphical abstract

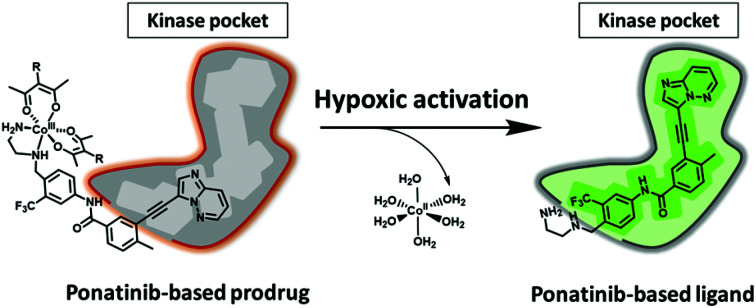

## Introduction

Tyrosine kinases play an essential part in the signal transduction pathways of cells, by catalyzing the transfer of γ-phosphate groups of adenosine triphosphate (ATP).^1^ Thus, they fulfil a crucial role in the control of *e.g.* cell growth, proliferation and differentiation. Dysregulations of these signaling processes have been linked to several diseases, including cancer initiation and progression.^2^ The development of tyrosine kinase inhibitors (TKIs) is a promising targeted therapeutic approach and resulted in the approval of respective antibodies and small molecule inhibitors.^3,4^ The latter competitively block the ATP-binding pockets in the active sites of tyrosine kinases.^3^ One representative of this substance class is ponatinib, a multi-kinase inhibitor, which targets among others the Abelson kinase (ABL), fibroblast growth factor receptor (FGFR), and platelet-derived growth factor receptor (PDGFR) families.^5–7^ Ponatinib is clinically approved as second line treatment for Philadelphia chromosome-positive chronic myeloid leukemia (Ph+ CML)/acute lymphoblastic leukemia (Ph+ ALL), especially in case of a BCR-ABLT315I mutation.^8–10^ Its anti-tumorigenic potential is also evaluated in several other cancer types,^11^ with the inhibition of FGFR being one major point of interest.^12,13^ However, despite the specific targeting of oncogene-dependent cancer cells, adverse effects, caused by the lack of distinction between healthy and cancerous tissue, could be frequently noticed.^14^ In a clinical phase III study severe arterial thrombotic events occured.^15,16^ As a result, the maximum applicable dose of ponatinib for clinical routine was reduced and further safety measures were included.^15,17^

**Fig. 1:**
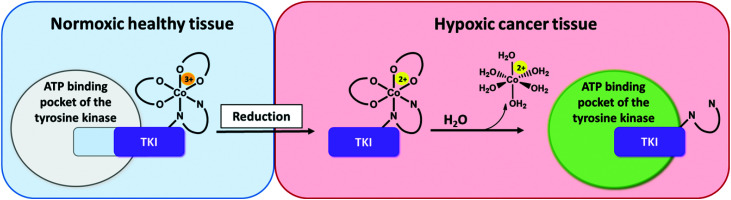
Proposed mode of action of hypoxia-activated cobalt(iii) prodrugs. **Normoxic, healthy tissue (left side):** The prodrug is inactive. Due to its bulkiness, the cobalt(iii) complex does not fit into the ATP-binding pocket of the respective tyrosine kinase. **Hypoxic cancer tissue (right side):** The drug is active. In the hypoxic tissue of the tumor, the cobalt(iii) complex is irreversibly reduced and the TKI ligand subsequently released able to inhibit the signaling processes of the target tyrosine kinases.

To optimize the therapeutic potential of ponatinib, a promising way could be the use of prodrug systems. In general, anticancer prodrugs consist of non-toxic, inactive compounds, which only release their active counterpart through specific activation in the tumor.^18^ Cancerous tissue exhibits certain features, such as a hypoxic environment, which can be exploited for this kind of approach.^19,20^ Hypoxia-activated prodrugs (HAPs) have been intensively explored over the past years for various substance classes with a focus on DNA alkylating agents.^21–23^ Although some of these compounds (*e.g.* tirapazamine, evofosfamide, apaziquone) have entered advanced clinical studies, none of them received approval so far.^24,25^ Surprisingly, until now this prodrug approach has been implemented only on a few TKI compounds,^26–28^ with TH-4000 as one of the first examples.^29^ Various chemical possibilities to exploit hypoxia are known with nitroimidazoles^27–29^ and cobalt(iii) metal complexes as important representatives.^26,30,31^ The mechanism of the latter is based on distinct differences in ligand lability of the cobalt(iii) (kinetically inert) and cobalt(ii) (kinetically labile) oxidation state.^32,33^ Due to its bulkiness, the intact cobalt(iii) complex should not be able to bind to the ATP binding pocket of the respective kinase (Fig. 1). In healthy tissue (normoxic conditions) the complex is stable, thus the release of the bioactive ligand is prevented.^34^

In contrast, in the hypoxic environment of a tumor, the inactive prodrug will be irreversibly reduced. This results in the dissociation of the complex, with formation of [Co(ii)(H_2_O)_6_]^2+^ and release of the bioactive ligand with subsequent binding to the kinase pocket (Fig. 1).

In such a prodrug approach, a well-balanced equilibrium is important: on the one hand the cobalt(iii) complex needs to have a high stability in healthy tissue, on the other hand the bioactive ligand should be efficiently released after reduction under hypoxic conditions. Seminal work of Ware *et al.* demonstrated that monodentate aziridine ligands could not sufficiently stabilized and therefore bidentate chelating ligands are preferred.^33^

**Fig. 2:**
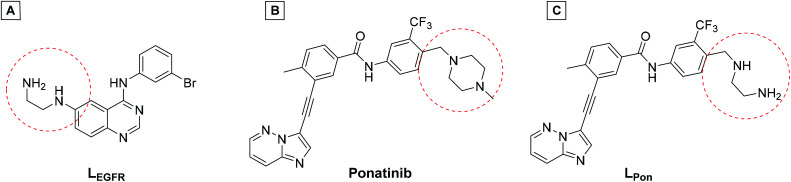
(A) Chemical structure of the previously synthesized EGFR inhibitor ligand (**LEGFR**) with a direct attachment of the ethylenediamine moiety to the aromatic ring system.^26^ (B) The clinically approved ponatinib (Iclusig®). (C) Newly developed ponatinib derivative (**LPon**), where the metal-chelating ethylenediamine moiety is separated from the aromatic ring system.

In a previous study of our group, we established the concept of cobalt(iii) prodrugs for EGFR-inhibitors, based on Erlotinib derivatives (**LEGFR**; Fig. 2).^26^ We observed that, due to the direct connection of the ethylenediamine unit to the quinazoline ring system, the reduction potential of the respective cobalt(iii) complex distinctly increased resulting in reduced stability in blood serum. In this study we focused on ponatinib, which is not only a highly active, clinically approved anticancer drug, but possesses also a –CH_2_-spacer between the solubilizing piperazine moiety and the aromatic ring system (**LPon**; Fig. 2). As ancillary ligands of the cobalt(iii) complexes, acetylacetone (acac) or methylacetylaceton (Meacac) were used. The novel compounds were evaluated for their fluorescence properties, electrochemical potential, kinetic reduction behavior and serum stability. Additionally, their fluorescence properties and the biological activity were investigated under normoxic *vs.* hypoxic conditions in an FGFR- or ABL1-driven cancer cell background. Finally, the *in vivo* antitumor activity of the novel complexes was evaluated in the respective xenograft mouse models.

## Results and discussion

### Docking studies

**Scheme 1:**
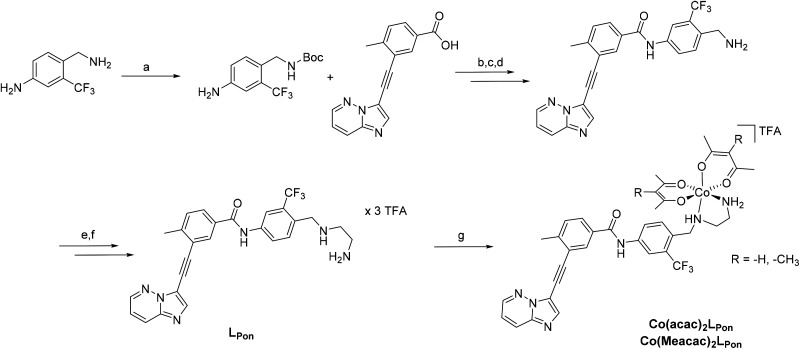
Synthesis of **LPon** and its cobalt(iii) complexes **Co(acac)2LPon** and **Co(Meacac)2LPon**. Reagents and conditions: (a) Di-*tert*-butyl dicarbonat, THF, 81%; (b) (COCl)_2_, abs. Toluol, NMM, DMAP, 68%; (c) 4 N HCl in dioxan/H_2_O, 95%; (d) NaHCO_3_ in EtOAc, 94%; (e) *N*-Boc-2-aminoacetaldehyde, sodium cyanoborhydride, abs. THF, molecular sieves (3–4 Å), 50%; (f) TFA in dichloromethane (ratio 1 : 1), 40%; (g) *in situ* deprotonation with NaOH, Na[Co(acac)_2_(NO_2_)_2_] or Na[Co(Meacac)_2_(NO_2_)_2_], activated charcoal in MeOH, 36% for **Co(acac)2LPon** and 31% for **Co(Meacac)2LPon**.

**Fig. 3:**
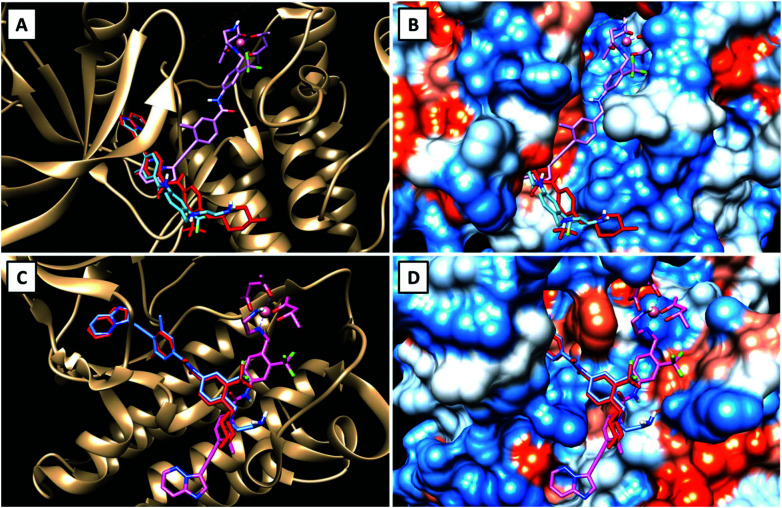
Visualizations of the best docking poses of **LPon** and **Co(acac)2LPon** in comparison to ponatinib with FGFR1 (A and B) (PDB ID: 4V04) as well as ABL1 (C and D) (PDB ID: 4WA9). Ponatinib is shown in red, **LPon** in blue and **Co(acac)2LPon** in pink.

To get a first indication if our proposed strategy is indeed promising, we performed docking studies of the novel ligand **LPon** and the two cobalt(iii) complexes **Co(acac)2LPon** and **Co(Meacac)2LPon** (Scheme 1) compared to the approved parental compound ponatinib. In general, the anticancer effect of TKIs is based on their strong intermolecular, non-covalent interactions with target-specific residues mainly consisting of hydrogen bonds, van der Waals, and Coulomb interactions.^35^ Consequently, molecular docking is a well-established tool in the field of TKI development.^36^ As kinases, two of the main targets of ponatinib were chosen, namely FGFR1 (PDB ID: 4V04) and ABL1 (PDB ID: 4WA9). The results indicated that the replacement of the piperazine moiety of ponatinib by an ethylenediamine group (**LPon**) did not change the binding mode in the pockets of FGFR1 (Fig. 3A and B) as well as ABL1 (Fig. 3C and D). In addition, the data of the complexes **Co(acac)2LPon** and **Co(Meacac)2LPon** convincingly showed that **LPon** attached to the cobalt(iii) core is unable to interact with the active sites of FGFR1 and ABL1. The steric effects of the complexation prohibited all crucial interactions with FGFR1 and ABL1 (Fig. 3 and S1†). Summarizing, the molecular docking studies showed that **LPon** is comparable to ponatinib in its kinase binding ability and that the cobalt(iii) complexes can be considered as promising prodrugs.

### Synthesis and characterization

To obtain a suitable metal-binding moiety for the complexation with a cobalt precursor, the piperazine was exchanged with an ethylenediamine unit (Scheme 1). To this end, the aliphatic amino group of the commercially available 4-(aminomethyl)-3-(trifluoromethyl)aniline was protected using di-*tert*-butyl dicarbonate. For the subsequent coupling reaction between the free aromatic amino group and purchased 3-(2-{imidazo[1,2-*b*]pyridazin-3-yl}ethynyl)-4-methylbenzoic acid, the carboxylic acid was first converted into the corresponding acyl chloride using oxalylchloride (COCl)_2_ in toluene.^37^ Subsequently, the amino group was deprotected using HCl, followed by neutralization of the formed HCl salt. Reductive amination with *N*-boc-(metylamino)-acetaldehyde and sodium cyanoborhydride yielded in the introduction of the protected ethylenediamine moiety. Finally, a second deprotection step with trifluoroacetic acid (TFA) and purification by reversed-phase HPLC generated the ethylenediamine-bearing ponatinib derivative **LPon** as three-fold TFA salt.

For the synthesis of the cobalt complexes, acetylacetone (acac) or methylacetylacetone (Meacac) were used as ancillary ligands, since these ligands can also distinctly influence the reduction potential of the complex.^33,38^ The cobalt(iii) complexes were synthesized by reaction of Na[Co(acac)_2_(NO_2_)_2_] or Na[Co(Meacac)_2_(NO_2_)_2_] with **LPon** (after the *in situ* deprotonation of the ligand with NaOH) in methanol and the presence of activated charcoal following a slightly modified procedure of Denny *et al.*^39^ Afterwards, the crude products were directly purified by reversed-phase HPLC (with the addition of 0.1% TFA to the eluents) leading to the respective TFA salts of **Co(acac)2LPon** and **Co(Meacac)2LPon**. Due to the two stereogenic centers (one from the propeller chirality of the complex itself and the other from the aliphatic NH-group of the ethylenediamine bridge), two isomers of the cobalt(iii) complexes are present. For **Co(acac)2LPon** we could separate the two isomers by adjusting the gradient of the preparative HPLC purification (Fig. S2†). All novel compounds were characterized by mass spectrometry as well as ^1^H and ^13^C one- and two-dimensional NMR spectroscopy and their purity was confirmed by elemental analysis. The amount of TFA was confirmed by ^19^F-NMR measurements *via* the ratio of the fluorine signals between **LPon** and TFA.

The ^1^H NMR spectra of the isomeric mixtures of the cobalt(iii) complexes [∼60% : 40% for **Co(acac)2LPon** and ∼70% : 30% for **Co(Meacac)2LPon**] show only one signal set in the aromatic area (with the exception of H23; see Experimental part), but two in the aliphatic area and of the –NH groups (Fig. S3 and S4†). This is in contrast to our previously synthesized cobalt(iii) complex with **LEGFR**, where all signals could be observed twice.^26^ An obvious explanation is that the conjugated system in the **LPon** ligand is much less extended compare to **LEGFR** (Fig. 2). A clear indication, that we were indeed able to lower the impact of the ring system on the chelating ethylenediamine moiety and *vice versa*.

**Fig. 4:**
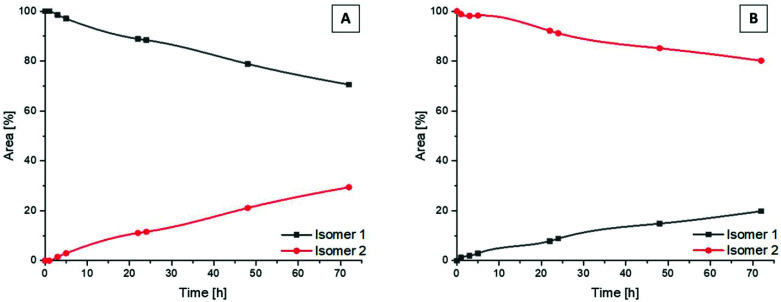
Kinetic behavior of the isomers of **Co(acac)2LPon** incubated in PBS at 37 °C (pH 7.4, 10 mM) and monitored by HPLC. Depicted is the conversion from pure isomer 1 (A) and pure isomer 2 (B) into the respective mixtures over a period of 72 h.

Following the successful isolation of the two isomers of **Co(acac)2LPon**, we studied their interconversion behavior in phosphate buffered saline (PBS) (pH 7.4, 10 mM) incubated at 37 °C. An interconversion is possible due to the chiral N of the ethylenediamine moiety, which can detach, invert and bind again, resulting in the other diastereomer. The results showed that the conversion from isomer 1 into isomer 2 proceeded with ∼30% after 72 h (Fig. 4A). In contrast, the reverse reaction from pure isomer 2 into isomer 1 yielded in only ∼20% conversion after 72 h (Fig. 4B). A higher kinetic stability of isomer 2 is also supported by data after 9 days, which reveal that initial pure isomer 1 is converted to a racemic mixture with isomer 2 (50%:50%). In case of pure isomer 2 still a ratio of ∼60 : 40 is present after 9 days. At the same time, these experiments revealed that the complexes are highly stable in aqueous solution at pH 7.4 without any release of the ponatinib-like ligand. Because of the slow conversion rates the pure isomers of **Co(acac)2LPon** were also biologically investigated (*vide infra*).

### Fluorescence properties

**Fig. 5:**
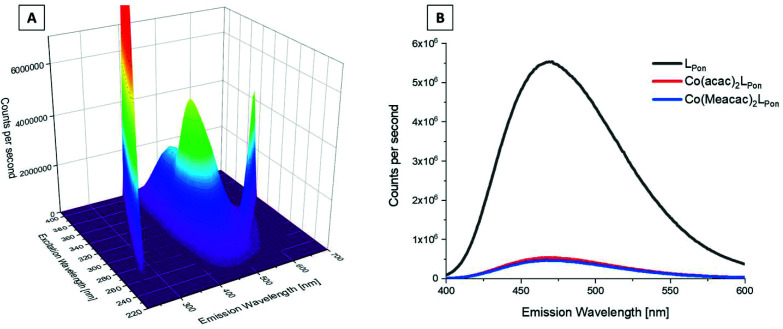
Fluorescence properties of investigated ponatinib derivatives. (A) 3D full excitation–emission landscape of **LPon** (Rayleigh scattering of 1^st^ and 2^nd^ order appear as diagonal ridges). (B) Fluorescence emission spectra at *λ*_ex_ = 320 nm of **LPon**, **Co(acac)2LPon**, and **Co(Meacac)2LPon**. Measurements were performed in PBS at pH = 7.40 [conc. ligand/complex = 15 μM; *T* = 25.0 °C].

We could recently show that ponatinib possesses distinct fluorescence properties with an emission maximum at 470 nm when irradiated at 320 nm (measured in PBS pH = 7.40, concentration = 15 μM).^40^ Therefore, we also analyzed the fluorescence of the novel ligand **LPon** and its cobalt(iii) complexes under the same conditions. The 3D spectrum of **LPon** showed a maximum and intensity similar to ponatinib (*λ*_em_ = 470 nm at *λ*_ex_ = 320 nm) (Fig. 5A). In contrast, for the cobalt(iii) complexes a strongly quenched fluorescence in PBS was observed (Fig. 5B). This phenomenon can be explained by the metal center, which influences the conversion of singlet-excited states to triplet-excited states with extremely fast intersystem crossing rates (typical lifetimes are in the fs scale). Hence, ligand-based fluorescence is often difficult to observe in a metal coordinated form.^41^ However, these distinct differences in the fluorescence properties between free ligand and cobalt(iii) complex can be exploited for stability studies in cell culture (*vide infra*).

### Cyclic voltammetry

**Fig. 6:**
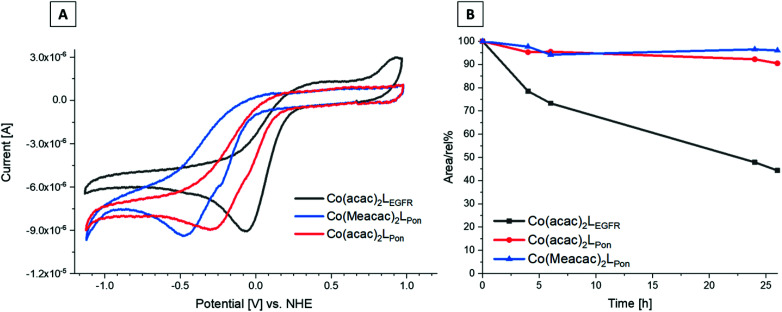
(A) Cyclic voltammograms of **Co(acac)2LPon**, **Co(Meacac)2LPon** and **Co(acac)2LEGFR** in DMF (1.5 mM complex, *I* = 0.2 M [*n*-Bu_4_N][BF_4_], scan rate of 100 mV s^−1^, 25.0 °C). (B) Stability measurements of **Co(acac)2LPon**, **Co(Meacac)2LPon** and **Co(acac)2LEGFR** incubated in pure FCS at 37 °C (pH 7.4, 150 mM phosphate buffer) analyzed by HPLC-MS over a time period of 26 h. The *y*-axis shows the relative ratio of the integrated peak areas of the intact complex over time (in percent) compared to the area at the starting point (0 h).

Since the redox potential is a crucial characteristic for the activation of cobalt(iii)-based prodrugs systems, we investigated the novel complexes by cyclic voltammetry in comparison to **Co(acac)2LEGFR**. Measurements were performed in dimethylformamide (DMF) (+0.2 M [*n*-Bu_4_N][BF_4_]) at a scan speed of 100 mV s^−1^. The voltammograms revealed a single irreversible cathodic peak, which can be assigned to the reduction of cobalt(iii) to cobalt(ii) (Fig. 6A). Even at a higher scan speed of 1000 mV s^−1^, the redox processes were still completely irreversible for all complexes (Fig. S5†).

The data clearly showed that the “separation” of the chelating ethylenediamine moiety from the ring system in case of the ponatinib complex **Co(acac)2LPon** resulted in the desired strongly decreased cathodic peak potential at −315 mV *vs.* NHE, compared to the directly attached EGFR-inhibitor reference **Co(acac)2LEGFR** at −57 mV *vs.* NHE. Methylation of the ancillary acetylacetone ligands in **Co(Meacac)2LPon** further shifted the potential to −442 mV *vs.* NHE. This in line with literature data of other cobalt(iii) prodrugs systems.^33,38^ Investigation of the same complexes in a DMF/H_2_O (7 : 3 v/v) mixture, resulted in similar shifts (Fig. S6†). In all measurements completely irreversible reduction processes were observed, regardless of the solvent or scan speed (100–1000 mV s^−1^). This is of interest, since the proposed mechanism for cobalt(iii) prodrugs is often associated with a reversible redox behaviour of these complexes. The frequently proposed model suggests that under normoxic conditions in healthy tissue the reduced cobalt(ii) complex will immediately be re-oxidized by oxygen to the inert cobalt(iii) complex and thus the release of the bioactive ligand is prevented.^34,42,43^ Interestingly, for most cobalt(iii) prodrugs reported in literature irreversible redox processes in aqueous solutions could be observed, which contradicts this hypothesis.^44–47^

Notably, pulse radiolysis studies of cobalt(iii) nitrogen mustard complexes indeed suggested that the re-oxidation rate under normoxia is too slow and the cobalt(iii) complexes rather compete with molecular oxygen for one-electron reductants.^48^ Nevertheless, different cobalt(iii) complexes with irreversible electrochemical behavior exhibited strong hypoxia-dependent activity against cancer cells *in vitro* and *in vivo*, proving the potential of such complexes.^26,45^

### Serum stability measurements

Recently, we could show that even the EGFR-inhibitor cobalt(iii) complex **Co(acac)2LEGFR** (with the highest redox potential) is completely stable in the presence of the natural low-molecular weight reducing agents ascorbic acid, glutathione or reduced nicotinamide adenine dinucleotide (NADH).^38^ Therefore, we only investigated the stability of the novel complexes in fetal calf serum (FCS). As reference again **Co(acac)2LEGFR**^26^ was included. The complexes were dissolved in 50 mM phosphate puffer and mixed 1 : 10 with fetal calf serum (FCS; buffered with 150 mM phosphate buffer to keep a stable pH of 7.4) to a final concentration of 50 μM. The samples were incubated at 37 °C and after 0, 2, 4, 6, 24 and 26 h extracted with acetonitrile and measured by HPLC-MS. Both ponatinib-derived complexes **Co(acac)2LPon** and **Co(Meacac)2LPon** were highly stable in FCS with ∼90% intact complex after 26 h (Fig. 6B). In contrast, the reference **Co(acac)2LEGFR** showed less than 50% remaining complex after the same incubation time, in line with our previous results.^38^ This indeed confirms, that by avoiding the direct attachment of the ethylenediamine moiety to an aromatic ring system, complexes with much higher stability can be generated. Since no distinct difference in stability between **Co(acac)2LPon** and **Co(Meacac)2LPon** within 26 h could be observed, measurements were repeated up to 72 h. Now the slightly increased stability of **Co(Meacac)2LPon** at ∼80% of intact compound compared to **Co(acac)2LPon** at ∼70% could be uncovered (Fig. S7†).

## Biological investigations

### Hypoxia-inducible intracellular ligand release

**Fig. 7:**
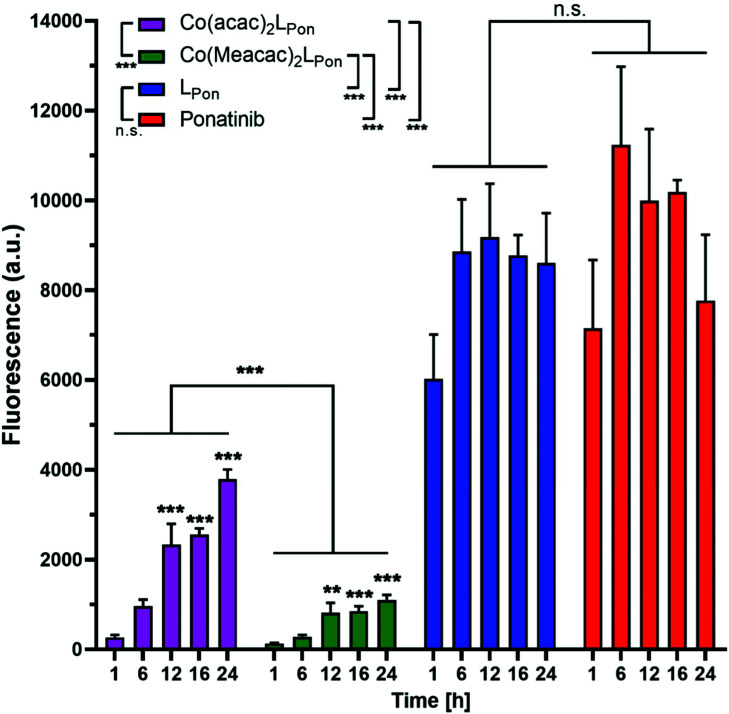
Fluorescence-based evaluation of **LPon** release from **Co(acac)2LPon** and **Co(Meacac)2LPon** in K-562 cells under normoxic cell culture conditions by flow cytometry. Cells were incubated with 10 μM of the respective compounds for the indicated exposure times and mean fluorescence intensities were determined by flow cytometry (BD LSRFortessa X-20, pacific blue filter). Fluorescence intensities were normalized by subtracting the auto fluorescence of untreated cells. Data are given in arbitrary units (a.u.) as means ± SEM of five independent experiments. Statistical significance was calculated by two-way ANOVA with *p* < 0.05(*); <0.01 (**); <0.001 (***).

Prior to the evaluation of the anticancer activity of our novel derivatives in cell culture models, we examined the stability of **Co(acac)2LPon** and **Co(Meacac)2LPon** in the presence of cells under normoxic conditions. The stability was determined by the appearance of **LPon**-associated fluorescence (*vide supra*) in BCR-ABL-driven K-562 leukemia cells *via* flow cytometry. As shown in Fig. 7, both ponatinib and **LPon** were taken up rapidly and efficiently by K-562 cells with no significant increase in cell-associated fluorescence from 1 h exposure onwards. In contrast, exposure of K-562 cells to equimolar concentrations of both cobalt(iii) complexes resulted in distinctly reduced cell-associated fluorescence levels, proving the pronounced stability of the complexes even in the presence of cells. Despite significant **LPon**-release from the two complexes over time (up to 24 h), in case of **Co(Meacac)2LPon** cell-associated fluorescence remained more than 7.5-fold lower after 24 h as compared to the free ligand. Moreover, the fluorescence intensities of **Co(Meacac)2LPon** were significantly reduced as compared to **Co(acac)2LPon**, well in agreement with the higher reduction potential of the latter complex.

**Fig. 8:**
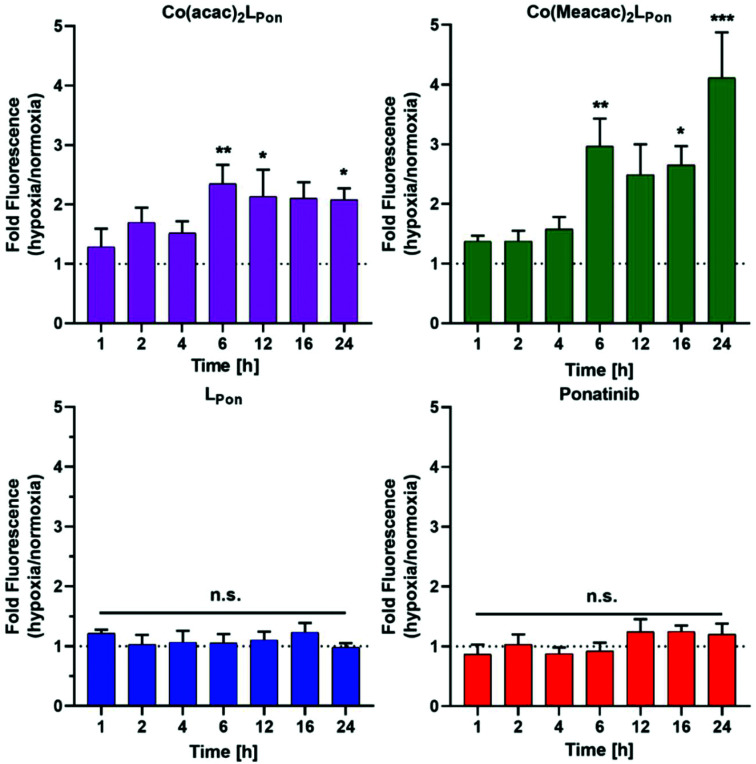
Impact of hypoxia on cell-associated **LPon**-fluorescence from **Co(acac)2LPon** and **Co(Meacac)2LPon**. K-562 cells were incubated with 10 μM of compounds for the indicated time points under hypoxic and normoxic conditions and fluorescence intensities were determined by flow cytometry (BD LSRFortessa X-20, pacific blue filter). Fluorescence intensities were determined as described under Fig. 7 and mean fluorescence intensities under hypoxic cell culture conditions were normalized to the respective normoxic conditions. Data are given as means ± SEM of five independent experiments. Statistical significance was calculated by one-way ANOVA with Dunnett multiple comparison test with *p* < 0.05(*); <0.01 (**); <0.001 (***).

Next, the impact of hypoxia on the stability of our novel derivatives was evaluated by monitoring intracellular fluorescence intensities under normoxic (21% O_2_) *vs.* hypoxic (0.1% O_2_) conditions. In general, only in case of the cobalt(iii) complexes, but not the free ligand **LPon** and ponatinib, a significant impact of oxygen conditions on cell-associated fluorescence was observed (Fig. 8). Hypoxic fluorescence activation in case of both cobalt(iii) complexes was time-dependent, reaching significance after 6 h of compound incubation and resulting in 2- to 4-fold higher intensities. The activation ability tended to be higher in the more stable **Co(Meacac)2LPon** complex, probably related to the lower spontaneous reduction under normoxic conditions (compare Fig. 7).

The enhanced stability of **Co(acac)2LPon** under normoxia and its hypoxia-induced ligand release was subsequently confirmed by fluorescence microscopy. Fluorescence intensities indicated a ∼2.8-fold decreased stability of the cobalt(iii) complex under hypoxic *vs.* normoxic cell culture conditions. In case of the free ligand **LPon**, fluorescence intensities remained unaffected by different oxygen levels (Fig. S9†). These findings are well in agreement with the flow cytometric analysis of the intracellular ligand release, demonstrating reduced stability, *i.e.* enhanced ligand release of the cobalt(iii) complexes in hypoxic *vs.* normoxic environments.

### Kinase screening

To investigate how the structural changes of the novel derivative affect its ABL1 and FGFR1 kinase-inhibitory potential in comparison to ponatinib, both substances were tested in cell-free kinase inhibition assays in the presence of a 10-fold excess of ATP (Fig. S8†). The IC_50_ values for ponatinib, which were 1.75 and 5.54 nM against ABL1 and FGFR1, are in the same range as reported in literature.^10^

In comparison, the IC_50_ values of **LPon** against ABL1 and FGFR1 was 1.93 and 25.9 nM, respectively. Consequently, the ABL1 inhibition potential of our new ligand is comparable to ponatinib, whereas the FGFR inhibition is distinctly weaker by a factor of ∼5.

### Anticancer activity against ABL1- and FGFR-driven cancer cell models

**Fig. 9:**
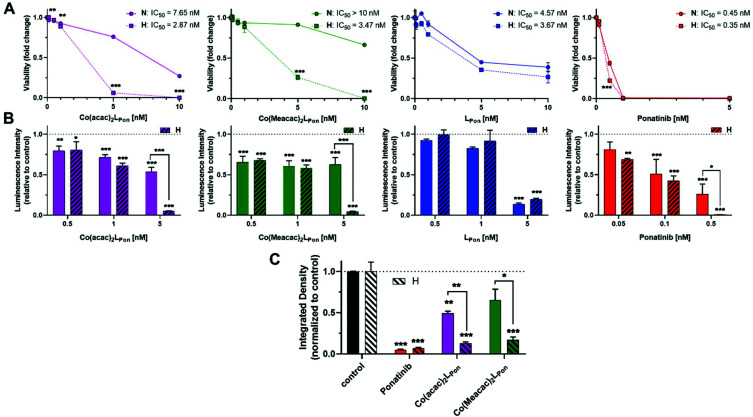
Anticancer activity of **Co(acac)2LPon**, **Co(Meacac)2LPon**, **LPon** and ponatinib against human cancer cell models under normoxic (*N*) and hypoxic (*H*) conditions. (A) BCR-ABL-positive K-562 leukemic cell viability was measured by MTT vitality assay and (B) by luminescence assay based on ATP quantification (CellTiter-Glo) after 72 h. (C) Clonogenic cell growth of the FGFR3-driven UM-UC-14 urothelial cancer cell model determined by colony formation assay after 5 d of incubation. Data are given as means ± SD of one representative experiment performed in triplicates. Statistical significance was calculated by two-way ANOVA with Sidak multiple comparison test with *p* < 0.05(*); <0.01 (**); <0.001 (***).

As a next step, the impacts of the novel derivatives on cell viability of the leukemia cancer cell line K-562 (BCR-ABL-dependent)^49^ and the urothelial cancer cell line UM-UC-14 (FGFR3-dependent)^50^ were evaluated under normoxic and hypoxic conditions. In general, a 72 h continuous cell exposure to all investigated compounds resulted in a dose-dependent reduction of cell vitality in the tested cell models under both oxygen conditions as determined by two independent methods, *i.e.* MTT assay (Fig. 9A) and ATP-based viability quantification (Fig. 9B).

Treatment of BCR-ABL-driven K-562 cells with ponatinib resulted in a pronounced reduction of cell vitality with IC_50_ in the nanomolar range. Notably, the anticancer activity of ponatinib was distinctly stronger as **LPon**. As in the above-mentioned cell-free kinase assay, IC_50_ values of both compounds against ABL1 were comparable (compare Fig. S8†), a reduced cellular uptake of **LPon** as compared to the more lipophilic ponatinib might be expected (calculated logP values^51^: ponatinib = 4.42; **LPon** = 3.39). Both cobalt(iii) complexes were clearly less active as compared to the free ligand under normoxic conditions with **Co(Meacac)2LPon** showing the highest IC_50_ value. Anticancer activities of ponatinib and **LPon** were only marginally altered under hypoxia, reaching significance only at a concentration of 0.5 nM ponatinib with both assays (Fig. 9A). In contrast, reduction of oxygen to 0.1% massively increased the anticancer activity of **Co(acac)2LPon** and **Co(Meacac)2LPon** against K-562 cells at a concentration range between 5 and 10 nM.

In FGFR3-dependent UM-UC-14 cells a comparable activity pattern was observed but with IC_50_ values in the low micromolar range (Fig. S10†). Again, target cells could be strongly sensitized by oxygen reduction against both cobalt(iii) complexes, but only marginally to ponatinib. The sensitizing effect in this cell model was more pronounced for **Co(acac)2LPon***vs.***Co(Meacac)2LPon** reflecting the differences in the reduction potentials. Purification of the novel compounds was performed in the presence of 0.1% TFA, leading to the respective TFA salts. MTT assays with TFA in UM-UC-14 cells elucidated, that TFA up to 25 μM had no impact on cell viability (Fig. S11†).

In order to investigate the impact of long-term oxygen restriction, clonogenic assays were performed in the FGFR3-dependent UM-UC-14 cell model (Fig. 9C). Under normoxic conditions both cobalt(iii) complexes inhibited clonogenic cell growth by approximately 50% only, whereas ponatinib by 95%. In contrast, under hypoxic conditions all three compounds were comparably active with more than 80% inhibition of clone formation.

As mentioned above, two stereoisomers of **Co(acac)2LPon** could be isolated. To clarify, whether there are differences in their anticancer activity, they were analyzed (in addition to the isomeric mixture) in UM-UC-14 cells by MTT assay. The impact on cell viability was moderately stronger for isomer 1 as compared to isomer 2 at normoxic conditions. Accordingly, values for the isomeric mixture positioned intermediate. As expected, both stereoisomers and the isomeric mixture were distinctly activated by hypoxic conditions. The moderately higher activity of isomer 1 remained also under hypoxic conditions (Fig. S12A†). Comparable effects were found in K-562 by luminescence assay-based ATP quantification (Fig. S12B†). Since no remarkable difference in anticancer activity could be observed for the pure isomers, the isomeric mixture was used for all other experiments.

It is known from literature that cobalt(ii) ions (which are released after reduction of the cobalt(iii) complexes) can cause cytotoxicity^52,53^ and changes in the hypoxia inducible factor (HIF-1 alpha).^54^ In our previous work we therefore investigated the effect of CoCl_2_, and the two complexes [Co(ii)(acac)_2_en] and [Co(iii)(acac)_2_en]PF_6_ (“en” = ethylenediamine) without an EGFR-targeting moiety on two cancer cell lines (A431 and H1975).^26^ Notably, all compounds showed no significant cytotoxic activity under normoxic as well as hypoxic conditions. Consequently, a major contribution of cobalt(ii) ions on the anticancer activity of our prodrugs can be widely excluded. Concerning upregulation of HIF-1 alpha signalling, it should be considered that our metal complexes are active in the nanomolar to the low micromolar range, while in most papers using CoCl_2_ as hypoxia-mimicry, concentrations of 100 μM or even higher are used.^55^

### Impact of hypoxia on downstream signalling inhibition

**Fig. 10:**
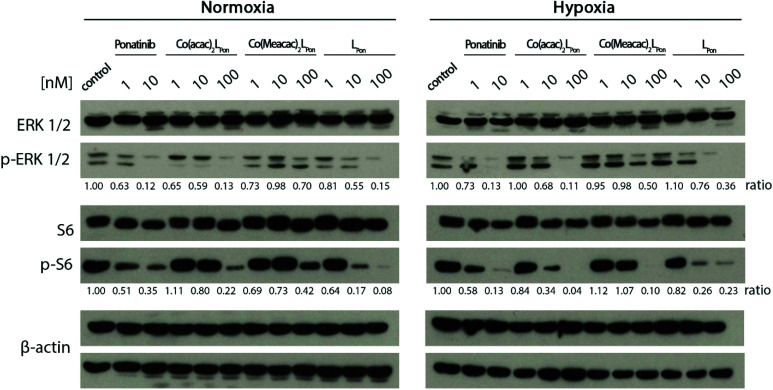
Impact of the cobalt(iii) complexes on the ERK1/2 and S6 under normoxic and hypoxic conditions. K-562 cells were treated with the compounds, after 12 h cell lysates were prepared and protein expression as well as phosphorylation levels of downstream pathways (p-ERK1/2 and p-S6) analyzed by western blotting. One representative experiment out of three is shown. The ratio of phosphorylated to total protein is given between the respective lanes.

Next, the impact of both cobalt(iii) complexes, **LPon** and ponatinib on the phosphorylation of the downstream targets ERK1/2 and S6 was evaluated under normoxic *vs.* hypoxic conditions by western blot analysis in BCR-ABL-driven K-562 cells (Fig. 10).

Generally, ERK1/2 phosphorylation, as readout of the MAPK pathway, tended to be enhanced under hypoxic conditions, while S6, as PI3K/AKT pathway indicator, tended to be reduced. Ponatinib and the free ligand **LPon** were equally active under the two oxygen conditions. However, comparable to the viability assays, the efficacy of **LPon** was lower as compared to ponatinib. The cobalt(iii) complexes were distinctly more active under hypoxic conditions, especially at the level of S6 phosphorylation. In this case, **Co(acac)2LPon** turned out to be equally active as compared to ponatinib. The activity of **Co(Meacac)2LPon** was generally weaker as compared to **Co(acac)2LPon**.

### *In vivo* anticancer activity

**Fig. 11:**
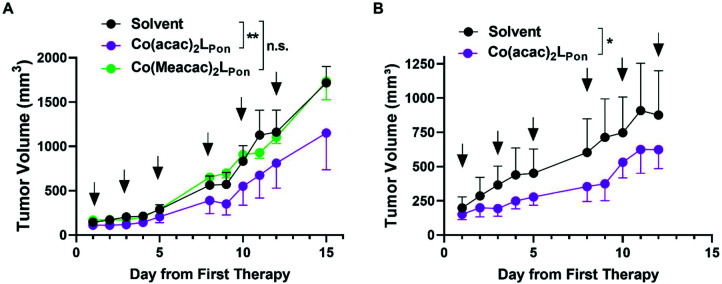
*In vivo* anticancer activity of the investigated cobalt(iii) complexes. (A) BCR-ABL-driven leukemic K-562 cells or (B) FGFR3-driven urothelial UM-UC-14 cells were injected s.c. into the right flank of male CB17/SCID mice (*n* = 4 animals per experimental group). When tumors were measurable (day 5 and day 7, respectively) compounds (10 mg kg^−1^ i.p.) were applied as indicated (black arrows). Tumor sizes were evaluated by caliper measurement. Data are given as means ± SEM. Statistical significance was calculated by two-way ANOVA with Sidak multiple comparison test with *p* < 0.05(*); <0.01 (**).

Based on this promising *in vitro* data, the antitumor activity of our novel cobalt(iii) complexes **Co(acac)2LPon** and **Co(Meacac)2LPon** was evaluated in xenograft mouse models. As a first *in vivo* experiment, BCR-ABL-driven K-562 cells were injected subcutaneously (s.c.) and treated intraperitoneally (i.p.) with the test compounds at 10 mg kg^−1^ or the respective amount of solvent three times a week, for two weeks. Treatment with our novel derivatives was well tolerated and even after repeated i.p. applications no substantial body weight loss was observed (Fig. S13A†). Administration of **Co(acac)2LPon** but not of the slower **LPon**-releasing **Co(Meacac)2LPon** complex significantly reduced tumor growth of K-562 xenografts as compared to solvent controls (Fig. 11). Consequently, anticancer activity of **Co(acac)2LPon** was also investigated against FGFR3-driven UM-UC-14 xenografts using the identical treatment regime. Again, administration of **Co(acac)2LPon** displayed significant reduction of tumor volumes compared to control, without significant impact on body weight (Fig. 11, S13B†).

## Conclusions

TKIs greatly improved cancer therapy since their first approval two decades ago. However, therapy resistance and severe side effects are limiting their clinical application. In this study, we developed the first ponatinib prodrugs with the aim to exploit the hypoxic environment present in the malignant tissue for cancer-specific drug activation. Molecular docking studies showed that both cobalt(iii) prodrugs synthesized are unable to interact with the active sites of FGFR1 and ABL1 kinases and that the bioactive ligand **LPon** has comparable kinase inhibitory abilities as ponatinib. Cell-free kinase inhibition assays confirmed binding of the free ligand to the targeted kinases ABL1 and FGFR1, in the case of ABL1 with affinities comparable to ponatinib. Furthermore, investigation of the fluorescence properties demonstrated, that the intensities of **LPon** were comparable to ponatinib, whereas **Co(acac)2LPon** and **Co(Meacac)2LPon** exhibited a strongly quenched fluorescence. Both derivatives were highly stable in pure FCS with ∼90% intact complex after 26 h. Nevertheless, according to cyclic voltammetry, **Co(Meacac)2LPon** exhibited a clearly lower reduction potential as compared to **Co(acac)2LPon**, suggesting higher stability and slower ligand release. This was confirmed, exploiting the distinct differences in the fluorescence properties between free ligand and cobalt(iii) complexes. Flow cytometry confirmed significantly enhanced spontaneous release of the bioactive ligand from **Co(acac)2LPon** as compared to **Co(Meacac)2LPon** under normoxic conditions, but potent release of both compounds under hypoxia. Moreover, reduced oxygen levels significantly increased inhibition of FGFR3-downstream signaling and improved the anticancer activity of both cobalt(iii) complexes against ABL- and FGFR-dependent human cancer cell models. Finally, **Co(acac)2LPon**, but not **Co(Meacac)2LPon** significantly reduced *in vivo* xenograft growth of the leukemic K-562 model. The *in vivo* activity of **Co(acac)2LPon** was confirmed in an FGFR3 mutation-dependent urothelial carcinoma xenograft background. This suggests, that especially in the leukemic background hypoxic conditions might be too weak to activate **Co(Meacac)2LPon** based on the lower reduction potential and, hence, higher stability. However, it needs to be considered that in the current study relatively small tumors were treated, due to a rapid tumor growth. Therefore, we currently aim to compare these drugs in slow growing cancer xenografts at later stages and to identify other solid tumor models, characterized by massive tumor hypoxia and driven by ponatinib targets. This will allow to further elucidate the interplay between cobalt(iii)-based prodrug systems and its reduction kinetics, molecular targets and cancer hypoxia.

## Experimental section

### Docking studies

The 3D structures of the molecules (**LPon**, **Co(acac)2LPon** and **Co(Meacac)2LPon**) have been drawn and optimized with Avogadro version 1.2.0,^56^ using Force Field GAFF (General AMBER Force Field).^57^ The molecules obtained are those with minimum energy. The crystal structures of FGFR1 (PDB ID: 4V04) and ABL1 (PDB ID: 4WA9) have been obtained from the RCSB Protein Data Bank.^58^ The *.pdb files from proteins were manipulated by elimination of adsorbed water and sulfur dioxide molecules, addition of missing hydrogen atoms, protonation of histidine residues, conversion of selenomethionines in methionines, addition and optimization of the side chain atoms. New *.pdb files were created of the proteins without the co-crystallized ligands and of the native ligands without the proteins using Chimera UCFS version 1.11.2.^59^ The *.pdbqt files of drugs and proteins were generated using AutodockTools-1.5.7rc.^60^ Ligand based molecular docking was performed with Autodock Vina 1.5 using ponatinib coordinates for FGFR1 and Axitinib for ABL1 to create the grid box.^61^ The coordinates of the grid box are: FGFR1 (*x*-size: 30, *x*-center: 27.814, *y*-size: 46, *y*-center: 5.016, *z*-size: 54, *z*-center: 16.789); ABL1 (*x*-size: 48, *x*-center: 7.819, *y*-size: 46, *y*-center: 156.948, *z*-size: 46, *z*-center: 37.479). Docking studies were performed in triplicates and the calculated RMSD value was always less than 1.5 Å.^62^

## Materials and methods

All solvents and reagents were obtained from commercial suppliers. They were, unless stated otherwise, used without further purification. Anhydrous MeOH and THF were bought from Fisher Chemicals over molecular sieves. The cobalt(iii) precursors Na[Co(acac)_2_(NO_2_)_2_] and Na[Co(Meacac)_2_(NO_2_)_2_] were obtained by following the protocol of Denny *et al.*^33^ For all HPLC measurements Milli-Q water (18.2 MΩ cm, Merck Milli-Q Advantage, Darmstadt, Germany) was used. Preparative RP-HPLC was performed on an Agilent 1200 Series system controlled by Chemstation software. As stationary phase a XBridge BEH C18 OBD Prep Column (130 Å, 5 μm, 19 mm × 250 mm) from Waters Corp., Massachusetts, USA, was used. The general procedure included a flow rate of 17.06 mL min^−1^, an injection volume of up to 10 mL and a column temperature of 25 °C. Milli-Q water and acetonitrile with addition of acids (0.1% TFA) were used as eluents unless stated otherwise. Elemental analyses were performed by the Microanalytical Laboratory of the University of Vienna on a PerkinElmer 2400 CHN Elemental Analyzer. The amount of TFA was also confirmed by ^19^F NMR spectra. Electrospray ionization (ESI) mass spectra were recorded on a Bruker Amazon SL ion trap mass spectrometer in positive and/or negative mode by direct infusion.

High resolution mass spectra were measured on a Bruker maXis™ UHR ESI time of flight mass spectrometer. Expected and experimental isotope distributions were compared. ^1^H and ^13^C NMR, one- as well as two-dimensional, spectra were recorded in *d*_6_-DMSO with a Bruker FT-NMR Avance III 500 MHz spectrometer at 500.10 (^1^H) and 125.75 (^13^C) MHz at 298 K. Chemical shifts (ppm) were referenced internal to the solvent residual peaks. For the description of the spin multiplicities the following abbreviations were used: s = singlet, d = doublet, t = triplet, q = quartet, m = multiplet.

### Synthesis

#### *tert*-Butyl (4-amino-2-(trifluoromethyl)phenyl)carbamate

4-Amino-2-trifluoromethylbenzyl amine (2.03 g, 1.0 eq.) was dissolved in THF (20 mL) at room temperature and di-*tert*-butyl dicarbonate (2.58 g, 1.1 eq.) was added to the solution. After stirring overnight, the solvent was removed and a reddish, viscous oil was obtained. The crude product was purified *via* column chromatography (hexane : ethyl acetate 3 : 2), resulting in yellow crystals. Yield: 2.51 g (81%). ^1^H NMR (500.1 MHz, DMSO-d_6_): *δ* 7.24 (t, 1H), 7.11 (d, *J* = 8.4 Hz, 1H), 6.85 (d, *J* = 2.3 Hz, 1H), 6.75 (d, *J* = 8.3 Hz, 1H), 5.44 (s, 2H), 4.12 (d, *J* = 5.8 Hz, 2H), 1.39 (s, 9H) ppm. MS: calcd for [C_13_H_17_F_3_N_2_O_2_–H]^−^: 289.12, found: 288.97.

#### *tert*-Butyl (4-(3-(imidazo[1,2-*b*]pyridazin-3-ylethynyl)-4-methylbenzamido)-2-(trifluoromethyl)benzyl) carbamate

3-(2-{Imidazo[1,2-*b*]pyridazin-3-yl}ethynyl)-4-methylbenzoic acid (0.95 g, 1 eq.) was suspended in toluene (abs., 90 mL) under argon atmosphere. Then, *N*-methylmorpholine (1.12 mL, 3 eq.) and (COCl)_2_ (1.79 mL, 1.1 eq., 2.0 M in dichloromethane) were added. The reaction was stirred at room temperature for 1.5 h, during which the solution become clear. The solvent was removed and the crude product was dried *in vacuo*, to remove any excess of (COCl)_2_. The solid was again suspended in toluene (abs., 90 mL) under argon atmosphere. *N*-Methylmorpholine (0.56 mL, 1.5 eq.), *tert*-butyl (4-amino-2-(trifluoromethyl)phenyl)carbamate (990 mg, 1 eq.) and 4-dimethylaminopyridine (4-DMAP) as catalyst (33 mg, 5 mol%), were added. The reaction mixture turned into a bright yellow color and was stirred at room temperature for 1 h, then refluxed overnight. The solvent was removed and the crude product dried *in vacuo*. After purification *via* column chromatography (pure ethyl acetate), the product was obtained as bright yellow crystals. Yield: 1.27 g (68%). ^1^H NMR (500.1 MHz, DMSO-d_6_): *δ* 10.58 (s, 1H), 8.73 (dd, *J* = 4.4 Hz *J* = 1.5 Hz, 1H), 8.29–8.23 (m, 2H), 8.21 (dd, *J* = 8.6 Hz, *J* = 1.8 Hz, 2H), 8.07 (d, *J* = 8.4 Hz, 1H), 7.95 (dd, *J* = 8.0 Hz, *J* = 1.9 Hz, 1H),7.56 (d, *J* = 8.2 Hz, 1H), 7.49 (dd, *J* = 11.9 Hz, *J* = 7.2 Hz, 2H), 7.40 (dd, *J* = 9.2 Hz, *J* = 4.4 Hz, 1H), 4.29 (d, *J* = 5.7 Hz, 2H), 2.61 (s, 3H), 1.41 (s, 9H) ppm. MS: calcd for [C_29_H_26_F_3_N_5_O_3_+Na]^+^: 572.1880, found: 572.1878. Anal. calcd for C_29_H_26_F_3_N_5_O_3_ (*M*_r_ = 549.55 g mol^−1^): C, 63.38; H, 4.77; N, 12.74. Found: C, 63.06; H, 4.82; N, 12.47.

#### (4-(3-(Imidazo[1,2-*b*]pyridazin-3-ylethynyl)-4-methylbenzamido)-2-(trifluoromethyl)phenyl) methanaminium chloride

*tert*-Butyl (4-(3-(imidazo[1,2-*b*]pyridazin-3-ylethynyl)-4-methylbenzamido)-2-(trifluoromethyl)benzyl) carbamate (1.26 g, 1 eq.) was dissolved in 1,4-dioxane (16.6 mL,) and 4 N HCl in dioxane/water (2.86 mL, 5 eq.) was added. The reaction was stirred at room temperature for about 3 h. The resulting white precipitate was filtered off, washed with 1,4-dioxane and diethyl ether. Yield: 1.12 g (94%). ^1^H NMR (500.1 MHz, DMSO-d_6_): *δ* 10.76 (s, 1H), 8.76 (dd, *J* = 4.4 Hz, *J* = 1.3 Hz, 1H), 8.52 (s, 3H), 8.34–8.27 (m, 3H), 8.25 (d, *J* = 1.5 Hz, 1H), 8.21 (d, *J* = 8.4 Hz, 1H), 7.99 (dd, *J* = 8.1 Hz, *J* = 1.7 Hz, 1H), 7.77 (d, *J* = 8.5 Hz, 1H), 7.58 (d, *J* = 8.1 Hz, 1H), 7.44 (dd, *J* = 9.2 Hz, *J* = 4.4 Hz, 1H), 4.15 (d, *J* = 5.4 Hz, 2H), 2.62 (s, 3H) ppm. MS: calcd for [C_24_H_18_F_3_N_5_O+H]^+^: 450.1536, found: 450.1533. Anal. calcd for C_24_H_18_F_3_N_5_O·HCl·H_2_O (*M*_r_ = 521.92 g mol^−1^): C, 55.23; H, 4.44; N, 13.42. Found: C, 54.91; H, 4.19; N, 13.25.

#### *N*-(4-(Aminomethyl)-3-(trifluoromethyl)phenyl)-3-(imidazo[1,2-*b*]pyridazin-3-ylethynyl)-4-methylbenzamide

(4-(3-(Imidazo[1,2-*b*]pyridazin-3-ylethynyl)-4-methylbenzamido)-2-(trifluoromethyl)phenyl) methanaminium chloride (240 mg, 1 eq.) was dissolved in deionized water and a saturated sodium bicarbonate solution was added until a slightly basic pH (7.5–8.0) was reached. The aqueous phase was extracted three times with ethyl acetate (3 × 50 mL). The organic layers were combined, dried over MgSO_4_ and *in vacuo*, resulting in a bright yellow powder. Yield: 183 mg (89%). ^1^H NMR (500.1 MHz, DMSO-d_6_): *δ* 10.55 (s, 1H), 8.73 (dd, *J* = 4.4 Hz, *J* = 1.5 Hz, 1H), 8.27 (dd, *J* = 9.2 Hz, 1.6 Hz, 1H), 8.24 (s, 1H), 8.20 (dd, *J* = 21.5 Hz, 2.0 Hz, 2H), 8.07 (dd, *J* = 8.5 Hz, 1.9 Hz, 1H), 7.95 (dd, *J* = 8.0 Hz, 1.9 Hz, 1H), 7.77 (d, *J* = 8.5 Hz, 1H), 7.56 (d, *J* = 8.1 Hz, 1H), 7.40 (dd, *J* = 9.2 Hz, 4.4 Hz, 1H), 3.84 (s, 2H), 2.61 (s, 3H) ppm. MS: calcd for [C_24_H_18_F_3_N_5_O+H]^+^: 450.15, found: 450.04.

#### *tert*-Butyl (2-((4-(3-(imidazo[1,2-*b*]pyridazin-3-ylethynyl)-4-methylbenzamido)-2-(trifluoromethyl) benzyl) amino) ethyl)carbamate

*N*-(4-(Aminomethyl)-3-(trifluoromethyl)phenyl)-3-(imidazo[1,2-*b*]pyridazin-3-ylethynyl)-4-methylbenzamide (470 mg, 1 eq.) was dissolved in abs. MeOH (abs., 13.5 mL) under inert conditions, molecular sieves (3–4 Å) and *N*-boc-2-aminoacetaldehyde (200 mg, 1.2 eq.) were added to the solution. The reaction was stirred at room temperature for 2 h. Subsequently, sodium cyanoborohydride (97 mg, 1.2 eq.) was added and the mixture was stirred overnight. The solvent was removed, the solid was dissolved in ethyl acetate (100 mL) and the molecular sieve was filtered off. The compound was extracted with saturated sodium bicarbonate solution (50 mL) and the organic phase was dried over MgSO_4_ and *in vacuo*. Yield of the crude product (according to HPLC): 50%.

^1^H NMR (500.1 MHz, DMSO-d_6_): *δ* 10.58 (s, 1H), 8.74 (dd, *J* = 4.5 Hz, *J* = 1.5 Hz, 1H), 8.28 (dd, *J* = 9.2 Hz, 1.6 Hz, 1H), 8.25 (s, 1H), 8.23 (d, *J* = 2.0 Hz, 1H), 8.22 (d, *J* = 2.0 Hz, 1H), 8.08 (dd, *J* = 8.5 Hz, 1.9 Hz, 1H), 7.96 (dd, *J* = 8.0 Hz, 1.9 Hz, 1H), 7.75 (d, *J* = 8.5 Hz, 1H), 7.56 (d, *J* = 8.1 Hz, 1H), 7.40 (dd, *J* = 8.9 Hz, 4.4 Hz, 1H), 6.80 (s, 1H), 3.84 (s, 2H), 3.09–3.02 (m, 2H), 2.62 (s, 3H), 2.60–2.55 (m, 2H), 1.38 (s, 9H) ppm. MS: calcd for [C_31_H_31_F_3_N_6_O_3_+H]^+^: 593.2482, found: 593.2485.

#### *N*-(4-(((2-Aminoethyl)amino)methyl)-3-(trifluoromethyl)phenyl)-3-(imidazo[1,2-*b*]pyridazin-3 ylethynyl)-4-methylbenzamide·3TFA (L_Pon_)

*tert*-Butyl (2-((4-(3-(imidazo[1,2-*b*]pyridazin-3-ylethynyl)-4-methylbenzamido)-2-(trifluoromethyl) benzyl) amino) ethyl)carbamate (400.3 mg, 1 eq.) was dissolved in dichloromethane (9 mL), TFA (9 mL) was added and the reaction was stirred at room temperature for 1 h. By adding diethyl ether to the orange solution, the product was precipitated as white solid and filtered off. The crude product (280 mg) was purified by preparative RP-HPLC (Waters XBridge C18 column on an Agilent 1200 Series system; H_2_O/ACN, both containing 0.1% TFA, 30.0–32.5% ACN gradient, 19 min per run) resulting in a beige, hygroscopic compound. Yield: 113 mg (40%). ^1^H NMR (500.1 MHz, DMSO-d^6^): *δ* 10.74 (s, 1H, H17), 9.62–9.09 (m, 2H, H26), 8.73 (s, 1H, H6), 8.32 (s, 1H, H19), 8.29–8.22 (m, 4H, H2, H4, H14, H24), 8.11–7.85 (m, 3H, H29), 7.96 (d, *J* = 8.0 Hz, 1H, H12), 7.78 (d, *J* = 8.5 Hz, 1H, H23), 7.58 (d, *J* = 7.6 Hz, H11), 7.40 (m, 1H, H5), 4.40–4.32 (m, 2H, H25), 3.34–3.28 (m, 2H, H27), 3.22–3.13 (m, 2H, H28), 2.62 (s, 3H, H15) ppm. ^13^C NMR (125.75 MHz, DMSO-d_6_): *δ* 164.8 (C16), 145.1 (C6), 143.8 (C10), 140.2 (C22), 139.7 (C3), 138.2 (C14), 132.1 (C23), 131.9 (C13), 130.2 (C24), 130.2 (C11), 128.5 (C12), 128.1 (C20), 126.2 (C4), 124.6 (C18), 123.6 (C2), 123.0 (C21), 121.8 (C9), 119.1(C5), 117.6 (C19), 111.6 (C1), 96.3 (C8), 81.2 (C7), 46.5 (C25), 44.3 (C27), 35.3 (C28), 20.4 (C15) ppm. MS: calcd for [C_26_H_23_F_3_N_6_O+H]^+^: 493.1958, found: 493.1962. Anal. Calcd for C_26_H_23_F_3_N_6_O·3TFA (*M*_r_ = 834.57 g mol^−1^): C, 46.05; H, 3.14; N, 10.07. Found: C, 46.28; H, 3.30; N, 9.89.
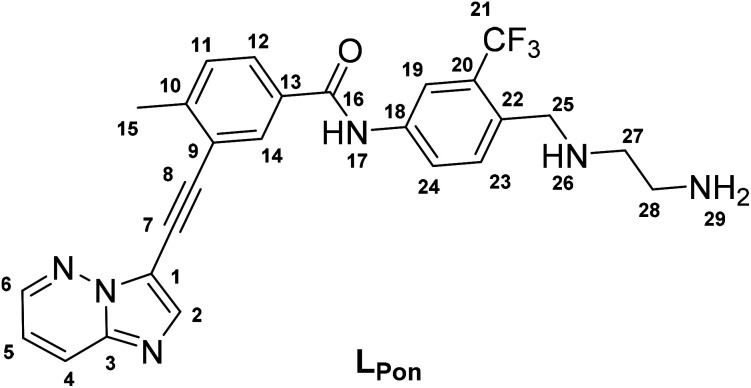


#### Bis(2,4-pentanedionato)*N*-(4-(((2-aminoethyl)amino)methyl)-3-(trifluoromethyl)phenyl)-3-(imidazo[1,2-*b*]pyridazin-3-ylethynyl)-4-methylbenzamide cobalt(iii) 2,2,2-trifluoroacetate (**Co(acac)2LPon**)

Na[Co(acac)_2_(NO_2_)_2_] (39 mg, 1 eq.) was suspended in MeOH (3.66 mL). **LPon** (88 mg, 1 eq.) was dissolved in MeOH (4.12 mL) and neutralized by NaOH (14 mg, 3 eq.). To the resulting yellow solution a spatula tip activated charcoal was added. The reaction was stirred for 2 h at room temperature. Afterwards the solution was filtered through a syringe filter, which was washed with small amounts of MeOH. The solvent was evaporated and the violet, crude product was dried *in vacuo* (130 mg). Purification was performed by preparative RP-HPLC (Waters XBridge C18 column on an Agilent 1200 Series system; H_2_O/ACN, both containing 0.1% TFA, isocratic gradient ACN 46%, 27 min per run) resulting in a violet, hygroscopic complex. Yield: 36 mg (36%). The ratio of the two isomers was 1 : 0.67.

Shifts of the main isomer; ^1^H NMR (500.1 MHz, DMSO-*d*_6_): *δ* 10.67 (s, 1H, H17), 8.73 (s, 1H, H6), 8.30–8.26 (m, 2H, H4, H19), 8.26–8.22 (m, 2H, H2, H14), 8.18–8.14 (m, 1H, H24), 7.97 (d, 1H, *J* = 9 Hz, H12), 7.78 (d, 1H, *J* = 8 Hz, H23), 7.58 (d, 1H, *J* = 9 Hz, H11), 7.43–7.39 (m, 1H, H5), 5.70 (s, 1H, CH, acac), 5.65 (s, 1H, CH, acac), 5.61 (s, 1H, H29), 5.26 (s, 1H, H26), 5.09 (s, 1H, H29), 3.89–3.79 (m, 2H, H25), 2.74–2.66 (m, 1H, H28), 2.63 (s, 3H, H15), 2.45–2.38 (m, 1H, H28), 2.55–2.45 (m, 1H, H27), 2.38–2.32 (m, 1H, H27), 2.15 (s, 3H, CH_3_, acac), 2.13–2.10 (s, 6H, CH_3_, acac), 2.06 (s, 3H, CH_3_, acac) ppm. ^13^C NMR (125.75 MHz, DMSO-*d*_6_): *δ* 189.9 (C_Q_, acac), 189.1 (C_Q_, acac), 188.9 (C_Q_, acac), 188.4 (C_Q_, acac), 164.7 (C16), 145.1 (C6), 143.7 (C10), 139.7 (C3), 139.3 (C18), 138.3 (C14), 133.7 (C23), 132.0 (C13), 130.2 (C11 + C19), 128.5 (C12), 128.4 (C22), 126.9 (C20), 126.1 (C4), 124.8 (C21), 123.2 (C24), 121.8 (C9), 119.1 (C5), 117.5 (C2), 111.6 (C1), 98.1 (CH, acac), 97.9 (CH, acac), 96.4 (C8), 81.2 (C7), 52.2 (C27), 47.1 (C25), 42.5 (C28), 26.5 (CH_3_, acac), 26.3 (CH_3_, acac), 26.1 (CH_3_, acac), 26.0 (CH_3_, acac), 20.4 (C15) ppm.

Shifts of the minor isomer; ^1^H NMR (500.1 MHz, DMSO-*d*_6_): *δ* 10.67 (s, 1H, H17), 8.73 (s, 1H, H6), 8.30–8.26 (m, 2H, H4, H19), 8.26–8.22 (m, 2H, H2, H14), 8.18–8.14 (m, 1H, H24), 7.97 (d, 1H, *J* = 9 Hz, H12), 7.91 (d, 1H, *J* = 8 Hz, H23), 7.58 (d, 1H, *J* = 9 Hz, H11), 7.43–7.39 (m, 1H, H5), 5.74 (s, 1H, CH, acac), 5.65 (s, 1H, CH, acac), 5.61 (s, 1H, H26), 5.48 (s, 1H, H29), 5.39 (s, 1H, H29), 3.63–3.58 (m, 1H, H25), 3.11–3.04 (m, 1H, H25), 2.63 (s, 3H, H15), 2.55–2.45 (m, 2H, H28), 2.45–2.38 (m, 2H, H28), 2.19 (s, 3H, CH_3_, acac), 2.13–2.10 (s, 6H, CH_3_, acac), 2.05 (s, 3H, CH_3_, acac) ppm. ^13^C NMR (125.75 MHz, DMSO-*d*_6_): *δ* 190.6 (C_Q_, acac), 189.9 (C_Q_, acac), 189.8 (C_Q_, acac), 189.5 (C_Q_, acac), 164.7 (C16), 145.1 (C6), 143.7 (C10), 139.7 (C3), 139.4 (C18), 138.3 (C14), 133.6 (C23), 132.0 (C13), 130.2 (C11 + C19), 128.5 (C12), 128.3 (C22), 126 126.6 (C20), 126.1 (C4), 124.8 (C21), 123.2 (C24), 121.8 (C9), 119.1 (C5), 117.5 (C2), 111.6 (C1), 98.1 (CH, acac), 98.0 (CH, acac), 96.4 (C8), 81.2 (C7), 50.9 (C27), 48.1 (C25), 42.3 (C28), 26.4 (CH_3_, acac), 26.3 (CH_3_, acac), 26.1 (CH_3_, acac), 26.0 (CH_3_, acac), 20.4 (C15) ppm.

MS: calcd For [C_36_H_37_F_3_N_6_O_5_Co]^+^: 749.2104, found: 749.2093. Anal. calcd for C_38_H_37_F_6_N_6_O_7_Co·0.6TFA·1.5H_2_O (*M*_r_ = 958.10 g mol^−1^): C, 49.14; H, 4.27; N, 8.77. Found: C, 48.84; H, 3.96; N, 8.53.

#### Bis(3-methyl-2,4-pentanedionato) *N*-(4-(((2-aminoethyl)amino)methyl)-3-(trifluoromethyl)phenyl)-3-(imidazo[1,2-*b*]pyridazin-3-ylethynyl)-4-methylbenzamide cobalt(iii) 2,2,2-trifluoroacetate (**Co(Meacac)2LPon**)

Na[Co(Meacac)_2_(NO_2_)_2_] (27.8 mg, 1 eq.) was suspended in MeOH (2.41 mL). **LPon** (88 mg, 1 eq.) was dissolved in MeOH (2.71 mL) and neutralized by NaOH (9.17 mg, 3 eq.). To the resulting yellow solution a spatula tip activated charcoal was added. The reaction was stirred for 2 h at room temperature. Afterwards the solution was filtered through a syringe filter, which was washed with small amounts of MeOH. The solvent was evaporated and the purple, crude product was dried *in vacuo* (85 mg). Purification was performed by preparative RP-HPLC (Waters XBridge C18 column on an Agilent 1200 Series system; H_2_O/ACN, both containing 0.1% TFA, isocratic gradient ACN 50%, 27 min per run) resulting in a violet, hygroscopic lyophilisate. Yield: 22 mg (31%). The ratio of the two isomers was 1 : 0.43.

Shifts of the main isomer; ^1^H NMR (500.1 MHz, DMSO-*d*_*6*_): *δ* 10.66 (s, 1H, H17), 8.73 (s, 1H, H6), 8.30–8.21 (m, 4H, H2, H4, H14, H19), 8.17–8.13 (m, 1H, H24), 7.97 (d, 1H, *J* = 9 Hz, H12), 7.75 (d, 1H, *J* = 8 Hz, H23), 7.58 (d, 1H, *J* = 9 Hz, H11), 7.43–7.39 (m, 1H, H5), 5.52–5.42 (m, 1H, H29), 4.99 (s, 1H, H29), 4.81 (s, 1H, H26), 3.94–3.84 (m, 2H, H25), 2.72–2.65 (m, 1H, H28), 2.63 (s, 3H, H15), 2.47–2.42 (m, 1H, H27), 2.44–2.38 (m, 1H, H28), 2.38–2.33 (m, 1H, H27), 2.24 (s, 3H, CH_3_, Meacac), 2.20 (s, 3H, CH_3_, Meacac), 2.15 (s, 3H, CH_3_, Meacac), 2.13 (s, 3H, CH_3_, Meacac), 1.92 (s, 3H, CH_3_, Meacac), 1.88 (s, 3H, CH_3_, Meacac) ppm. ^13^C NMR (125.75 MHz, DMSO-*d*_6_): *δ* 188.1 (2C_Q_, Meacac), 187.3 (C_Q_, Meacac), 186.9 (C_Q_, Meacac), 164.7 (C16), 145.1 (C6), 143.8 (C10), 139.7 (C3), 139.2 (C18), 138.3 (C14), 133.4 (C23), 132.0 (C13), 130.1 (C11 + C2), 128.5 (C12), 128.2 (C20), 127.3 (C22), 126.1 (C4), 124.8 (C21), 123.1 (C24), 121.9 (C9), 119.2 (C5), 117.5 (C19), 111.7 (C1), 100.8 (C_Q_, Meacac), 100.7 (C_Q_, Meacac), 96.4 (C8), 81.2 (C7), 52.1 (C27), 46.9 (C25), 42.4 (C28), 26.6 (CH_3_, Meacac), 26.5 (2CH_3_, Meacac), 26.2 (CH_3_, Meacac), 20.4(C15), 14.9 (CH_3_, Meacac), 14.8 (CH_3_, Meacac) ppm.

Shifts of the minor isomer; ^1^H NMR (500.1 MHz, DMSO-*d*_6_): *δ* 10.66 (s, 1H, H17), 8.73 (s, 1H, H6), 8.30–8.21 (m, 4H, H2, H4, H14, H19), 8.17–8.13 (m, 1H, H24), 7.97 (d, 1H, *J* = 9 Hz, H12), 7.89 (d, 1H, *J* = 8 Hz, H23), 7.58 (d, 1H, *J* = 9 Hz, H11), 7.43–7.39 (m, 1H, H5), 5.52–5.42 (m, 1H, H29), 5.32 (s, 1H, H29), 5.12 (s, 1H, H26), 3.54–3.49 (m, 1H, H25), 2.99–2.93 (m, 1H, H25), 2.72–2.65 (m, 1H, H28), 2.63 (s, 3H, H15), 2.47–2.42 (m, 1H, H27), 2.44–2.38 (m, 1H, H28), 2.38–2.33 (m, 1H, H27) 2.28 (s, 3H, CH_3_, Meacac), 2.23 (s, 3H, CH_3_, Meacac), 2.18 (s, 3H, CH_3_, Meacac), 2.11 (s, 3H, CH_3_, Meacac), 1.92 (s, 3H, CH_3_, Meacac), 1.87 (s, 3H, CH_3_, Meacac) ppm. ^13^C NMR (125.75 MHz, DMSO-*d*_*6*_): *δ* 188.7 (C_Q_, Meacac), 188.2 (C_Q_, Meacac), 187.3 (C_Q_, Meacac), 187.0 (C_Q_, Meacac), 164.7 (C16), 145.1 (C6), 143.8 (C10), 139.7 (C3), 139.3 (C18), 138.3 (C14), 133.3 (C23), 132.0 (C13), 130.1 (C11 + C2), 128.5 (C12), 127.0 (C22), 126.1 (C4), 128.4 (C20), 123.1 (C24), 121.9 (C9), 119.2 (C5), 117.5 (C19), 111.7 (C1), 101.0 (C_Q_, Meacac), 100.7 (C_Q_, Meacac), 96.4 (C8), 81.2 (C7), 50.7 (C27), 48.0 (C25), 42.0 (C28), 26.4 (CH_3_, Meacac), 26.3 (CH_3_, Meacac), 26.2 (2CH_3_, Meacac), 20.4 (C15), 14.9 (CH_3_, Meacac), 14.8 (CH_3_, Meacac) ppm.

MS: calcd for [C_38_H_41_F_3_N_6_O_5_Co]^+^: 777.2417, found: 777.2405. Anal. calcd for C_40_H_41_F_6_N_6_O_7_Co·TFA·H_2_O (*M*_r_ = 1022.75 g mol^−1^): C, 49.32; H, 4.34; N, 8.22. Found: C, 48.98; H, 4.19; N, 8.01.

### Interconversion of the isomers

For the interconversion measurements, a stock solution of the two isomers of **Co(acac)2LPon** (5 mM in water) was prepared. The final concentration was reached by 1 : 100 dilution with PBS (pH 7.4, 10 mM). The samples were incubated at 37 °C. Measurements were performed on a Dionex UltiMate 3000 RS UPLC system with a Waters Acquity UPLC® BEH C18 column (3 × 50 mm, pore size 1.7 μm). Water and acetonitrile (both containing 0.1% TFA) were used as eluents, an isocratic gradient of 45% acetonitrile within 8 min was applied.

### Fluorescence measurements

Fluorescence measurements were performed on a Horiba FluoroMax®-4 spectrofluorometer and the data was analyzed using the FluorEssence v3.5 software package. The tested solutions were dissolved immediately prior to analysis in PB (50 mM, pH 7.4) with a final concentration of 15 μM. Scans were run at room temperature with excitation and emission slit widths of 3 nm. Emission scans were run from 250–700 nm using an excitation wavelength of 320 nm. The 3D fluorescence spectra of **LPon** were determined at excitation wavelengths from 230–550 nm and emission was recorded within the range of 220–700 nm.

### Cyclic voltammetry

The compounds were dissolved in DMF (+0.2 M [*n*-Bu_4_N][BF_4_]) to obtain a final concentration of 1.5 mM. Electrochemical experiments were conducted on an EG&G PARC 273A potentiostat/galvanostat with a scan rate of 100–1000 mV s^−1^ at room temperature. Argon was bubbled through the solution before every measurement to remove oxygen. A three-electrode configuration cell was used with a glassy carbon electrode as working electrode, which was polished before every measurement. The reference electrode was Ag/Ag^+^/ACN (10 mM AgNO_3_) and the auxiliary electrode was a platinum wire. The potentials were referenced to an internal standard redox couple of ferrocenium/ferrocene and calculated to the NHE (*E*_1/2_ = +0.72 V *vs.* NHE).^63^ For the cyclic voltammetry measurements in DMF/H_2_O (7 : 3 v/v; aqueous phase was 10 mM phosphate buffer at pH 7.40 with 0.1 M KCl), a glassy carbon electrode as working electrode, a platinum auxiliary electrode, and an Ag/AgCl electrode containing 3.5 KCl were used. Redox potentials measured relative to the Ag/AgCl/KCl (3.5 M) reference electrode and converted to the NHE using +0.205 V. Measurements were at least repeated three times and the mean values were calculated.

### Serum stability measurements

For serum stability measurements, 135 μL FCS, buffered with 150 mM phosphate (Na_2_HPO_4_/NaH_2_PO_4_) to maintain a pH value of 7.4, were mixed with 15 μL of a 500 μM stock solution of the respective complex in 50 mM phosphate buffer to reach a final concentration of 50 μM. The samples were incubated at 37 °C. At different time points to 20 μL serum 40 μL of ACN were added. After vigorously shaking for 2 min the suspension was centrifuged at 6000 rpm for 10 min. The supernatant was taken with a syringe and directly measured *via* HPLC-MS. The samples were analyzed on an Agilent 1260 Infinity system using a Waters Atlantis T3 column (150 mm × 4.6 mm) coupled to a Bruker Amazon SL ESI-IT mass spectrometer. Water and acetonitrile (both containing 0.1% formic acid) were used as eluents with a gradient of 1–99% acetonitrile within 29 min.

## Biological methods

### Kinase screening

The ABL1 and FGFR1 kinase-inhibitory potentials of the novel derivative **LPon** in comparison to ponatinib were evaluated using the Select Screen® Biochemical Kinase Profiling Service at Life Technologies (ThermoFisher Scientific, Madison, USA). The test compounds were screened in a final concentration of 1% DMSO using the Z′-LYTE® Assay. Results are additionally depicted in Fig. S8.†

### Cell lines, cell culture conditions and drug treatments

All reagents were purchased from Sigma-Aldrich (St Louis, USA) unless specified otherwise. The human leukemia cell line K-562 (chronic myeloid leukemia; BCR-ABL-positive) and human urothelial cancer cell line UM-UC-14 (FGFR3-dependent), obtained from the American Type Culture Collection (ATCC) (Rockville, MD, USA), were cultured in RPMI 1640 (K-562) or MEM (Minimum Essential Medium Eagle) (UM-UC-14) supplemented with 10% fetal calf serum (PAA, Linz, Austria) at 37 °C, 5% CO_2_ in a humidified atmosphere. In case of hypoxic cell culture conditions, plated and treated cells were incubated at 37 °C, 5% CO_2_ and 0.1% O_2_ (ProOx Model C21 system, BioSpherix) in humidified incubators for the indicated time point before analysis. All investigated compounds were dissolved in dimethyl sulfoxide (DMSO) as 10 mM stock solutions and were stored at −20 °C. Dilutions in culture media supplemented with 10% fetal calf serum were made immediately before the experiments at the indicated concentrations. Corresponding dilutions of DMSO were used as untreated vehicle controls.

### Cytotoxicity assay

Cells were seeded (3–7 × 10^4^ cells per well) in 96-well plates and after 24 h recovery treated with increasing concentrations of compounds in triplicates. After 72 h drug exposure under normoxic or hypoxic cell culture conditions (0.1% O_2_) (ProOx Model C21 system, BioSpherix), the proportion of viable cells was determined by 3-(4,5-dimethylthiazol-2-yl)-2,5-diphenyltetrazolium bromide (MTT)-vitality assay (EZ4U, Biomedica, Vienna, Austria) or by luminescence assay based on adenosine triphosphate quantification (CellTiter-Glo, Promega, Madison, Wisconsin) following the manufacturer's recommendations. Absorbance was measured at 450 nm (at 620 nm as reference) (MTT) and luminescence after 1000 ms (CellTiter-Glo) at the Tecan infinite 200Pro (Zurich, Switzerland). MTT-derived cytotoxicity was expressed as IC_50_ values calculated from full dose–response curves using GraphPad Prism software (La Jolla, CA).

### Colony formation assay

UM-UC-14 cells were seeded at low densities of 3 × 10^3^ cells per well in 24-well plates in 500 μL and after 24 h recovery treated with 100 μL of increasing concentrations of compounds in duplicates. Following drug exposure time of 5 days under normoxic or hypoxic (0.1% O_2_) culture conditions, cells were washed with phosphate-buffered saline (PBS), fixed with ice-cold methanol for 30 min at 4 °C and stained with crystal violet. Digital photographs were taken using a Nikon D3200 camera and processed with ImageJ software. For quantification, crystal violet was eluted using 2% sodium dodecyl sulfate (SDS) and color absorbance was measured at 560 nm at the Tecan infinite 200Pro (Zurich, Switzerland). Values are given in arbitrary units (a.u.) as mean ± SD normalized to untreated control.

### Flow cytometry

Cells were seeded (1 × 10^6^ cells per well) in 96-well plates, dissolved compounds were added and cells were incubated under normoxic or hypoxic cell culture conditions. Samples were measured on a BD LSRFortessa X-20 cell analyzer high throughput sampler (HTS) (BD Biosciences, East Rutherford, NJ, USA). Compound fluorescence was detected using 405 nm excitation and pacific blue (450/50 nm) band pass emission filters. Data were analyzed using BD FACSDiva software and are given in arbitrary units (a.u.) as mean fluorescence intensities of cells normalized to the auto fluorescence of untreated control.

### Fluorescence microscopy

UM-UC-14 cells were seeded (2.5 × 10^5^ cells per well) in 12-well plates and after 24 h recovery treated with 10 μM of compounds. After 24 h incubation, the drug solutions were removed, cells were washed with PBS and microphotographs of the different treatments were taken using UV fluorescence microscopy (Nikon Eclipse Ti2 microscope with a DAPI filter and a high-pressure mercury lamp) and a 20× objective. The level of cellular fluorescence was determined from fluorescence microscopy images using ImageJ software.

### Western blot analysis

K-562 cells were plated (5 × 10^5^ cells per well) in 6-well plates and allowed to recover for 24 h. Subsequently, the cells were treated with the drugs in different concentrations. After 12 h cells were harvested, proteins were isolated, resolved by sodium dodecyl sulfate polyacrylamide gel electrophoresis (SDS/PAGE), and transferred onto a polyvinylidene difluoride membrane for western blotting. The following antibodies were used: ERK1/2 (p44/42) rabbit monoclonal antibody (mAb) (137F5, #4695), phospho-ERK1/2 rabbit mAb (Thr202/Tyr204) (20G11, #4376), phospho-S6 rabbit pAb (Ser240/244, #2215) (Cell Signaling Technology, Beverly, MA, USA), S6 mouse mAb (C-8. #sc-74459) (Santa Cruz Biotechnology, TX, USA) and β-actin mouse mAb (#A5441, Sigma). All primary antibodies were used in 1 : 1000 dilutions (in Tris-buffered saline containing 0.1% Tween20 (TBST) + 3% bovine serum albumin (BSA)). Secondary goat anti-mouse-IgG (Fc specific)-peroxidase antibody (#A0168, Sigma) and horseradish peroxidase(HRP)-labeled mouse anti-rabbit IgG (#sc-2357, Santa Cruz Biotechnology) were used at working dilutions of 1 : 10 000 (in TBST + 1% BSA).

### *In vivo* xenograft experiment

Eight-to nine-week-old male C.B.17/SCID mice were bread in-house (originally Harlan) and were kept in pathogen-free conditions and controlled environment with 12 h light–dark cycle. K-562 (5 × 10^6^ in 100 μL serum-free RMPI medium, 20% Matrigel) or UM-UC-14 (1 × 10^6^ in 100 μL serum-free RMPI medium) cells were injected into the right flank of male C.B.17/SCID mice. When all tumors were measurable (at day 5 and day 7, respectively), all animals were dosed i.p. with solutions (200 μL) containing **Co(acac)2LPon** or **Co(Meacac)2LPon** (10 mg kg^−1^, 200 μL per 20 g, dissolved in 50 mM PBS, 2% DMSO). The control groups received solvent alone (200 μL, 50 mM PBS, 2% DMSO). Compounds were dissolved in the respective amount of DMSO and then added to PBS buffer for the desired end concentration allowing application of 200 μL per 20 g and dosing using 30 gauze needles. Tumor growth was evaluated by daily recording of tumor size by caliper measurement. Animals were sacrificed by cervical dislocation. All procedures were performed in a laminar flow hood. The animal experiments were performed according to the regulations of the Ethics Committee for the Care and Use of Laboratory Animals at the Medical University Vienna (proposal number BMBWF-V/3b 2020-0.380.502).

### Statistical analysis

Data were analyzed using GraphPad Prism 8.0 software (GraphPad Software, Inc.) and are expressed as mean ± standard deviation (SD) of one independent experiment performed in triplicates, unless stated otherwise. One- or two-way analysis of variance (ANOVA) with multiple comparison test (Sidak, Bonferroni or Dunnett) was performed for statistical evaluation if not stated otherwise, *p*-values <0.05 were considered statistically significant [*p* < 0.05(*); <0.01 (**); <0.001 (***)].

## Conflicts of interest

There are no conflicts to declare.

## Supplementary Material

QI-008-D1QI00211B-s001
