# Supplementary material for: Development of a cobalt(iii)-based ponatinib prodrug system
Source: Inorg Chem Front. 2021 Mar 30;8(10):2468–85. doi: 10.1039/d1qi00211b (PMC8129988; doi:10.1039/d1qi00211b)
Supplement: QI-008-D1QI00211B-s001 [file QI-008-D1QI00211B-s001.pdf]

Electronic Supplementary Material (ESI) for Inorganic Chemistry Frontiers.
This journal is © the Partner Organisations 2021

Supplementary material

Development of a cobalt(III)-based ponatinib prodrug system

Marlene Mathuber[a], Michael Gutmann[b], Mery La Franca[b,c], Petra Vician[b], Anna Laemmerer[b,d], Patrick Moser[b], Bernhard K. Keppler[a,d], Walter Berger[b,d]*and Christian R. Kowol[a,d]*

[a] Institute of Inorganic Chemistry, Faculty of Chemistry, University of Vienna, Waehringer Straße 42, 1090 Vienna, Austria

[b] Institute of Cancer Research and Comprehensive Cancer Center, Medical University of Vienna, Borschkegasse 8A, 1090 Vienna, Austria

[c] Department of Biological, Chemical and Pharmaceutical Sciences and Technologies, University of Palermo, via Archirafi 32, 90123 Palermo, Italy

[d] Research Cluster ''Translational Cancer Therapy Research'', University of Vienna and Medical University of Vienna, 1090 Vienna, Austria

* Corresponding authors. E-mail address: christian.kowol@univie.ac.at; walter.berger@meduniwien.ac.at

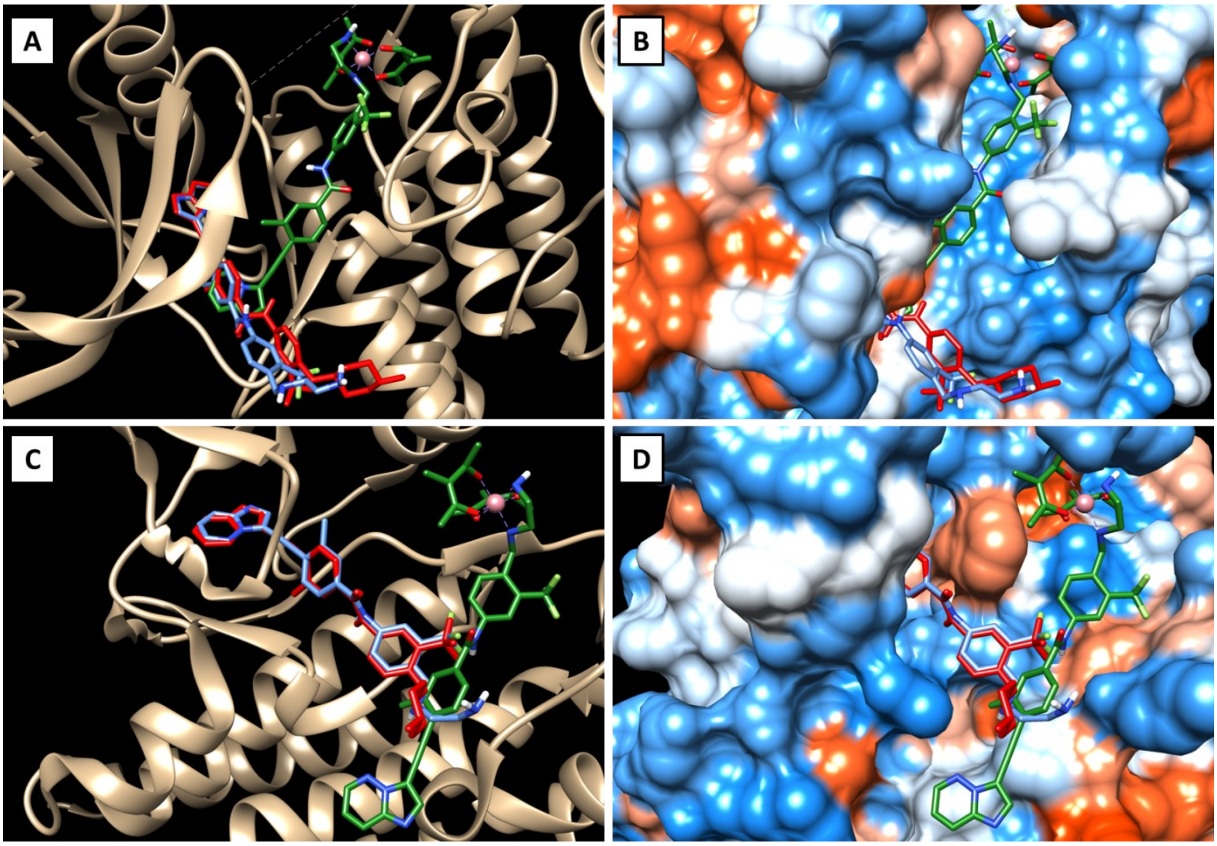

Figure S1: Visualizations of the best docking poses of $\mathbf{L_{Pon}}$ and $\mathbf{Co(Meacac)_2L_{Pon}}$ in comparison to ponatinib with FGFR1 (A and B) (PDB ID: 4V04) as well as ABL1 (C and D) (PDB ID: 4WA9). Ponatinib is shown in red, $\mathbf{L_{Pon}}$ in blue and $\mathbf{Co(Meacac)_2L_{Pon}}$ in green.

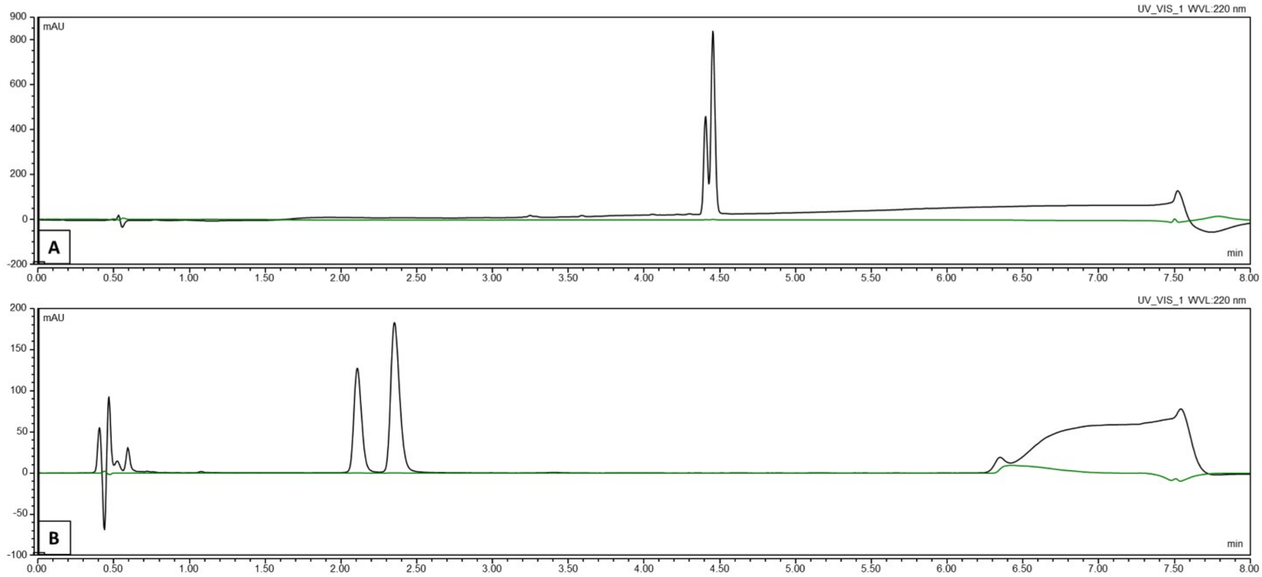

Figure S2: HPLC measurements of $\mathbf{Co(acac)_2L_{Pon}}$: A) Gradient from 5-95 % ACN (+0.1 % TFA), both isomers are visible, but strongly overlapping. B) Adjusted gradient 45 % ACN (+0.1 % TFA) isocratic, essential for separation via preparative HPLC.

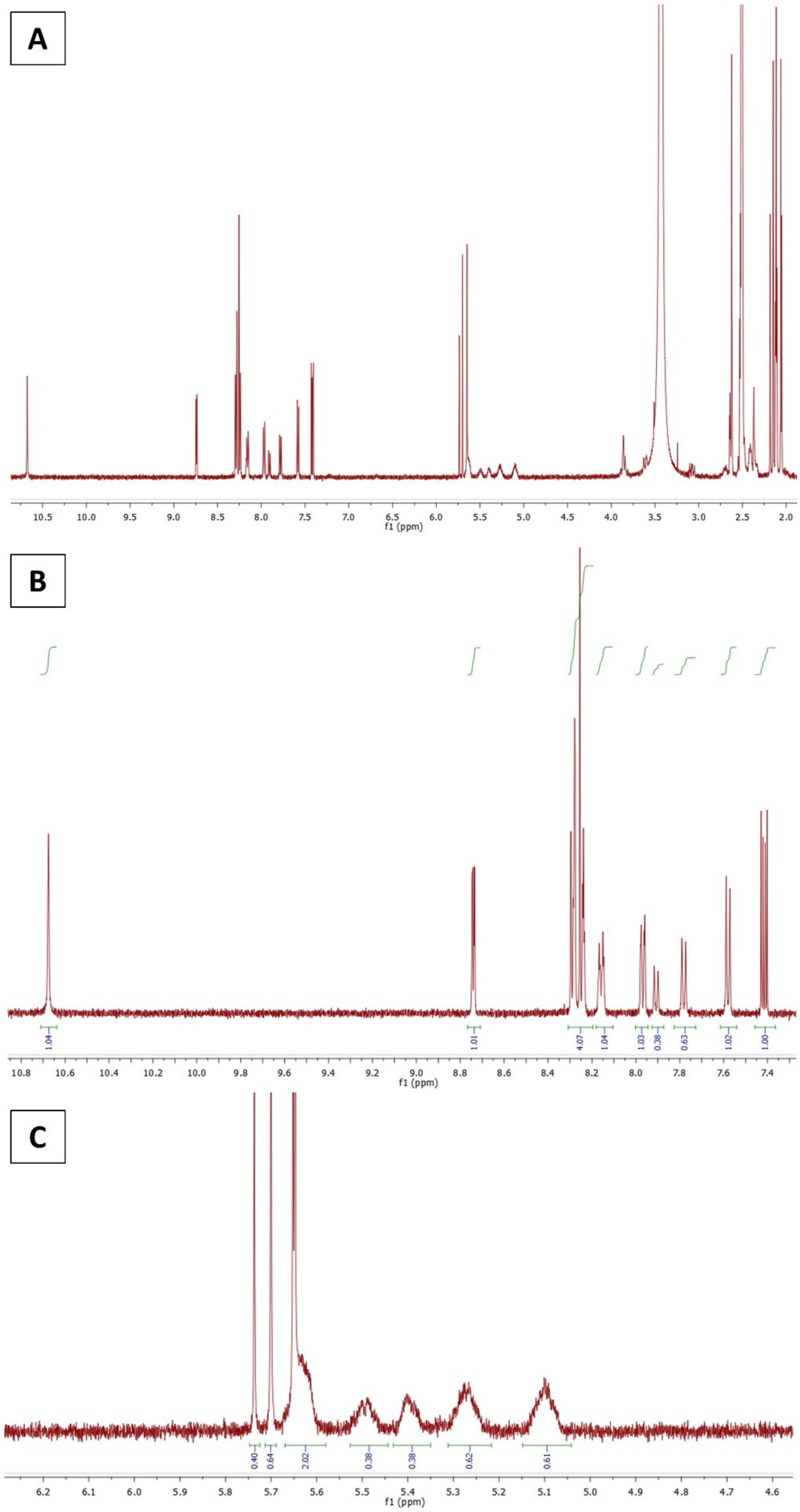

Figure S3: ^{1}H-NMR spectrum of **$Co(acac)_2L_{Pon}$,** showing (A) the whole-range, (B) the aromatic area from ~7–11 ppm and (C) the aliphatic area from ~4–6 ppm . Two sets of signals can be observed, but only in the aliphatic region and for the proton at 7.78/7.91 ppm.

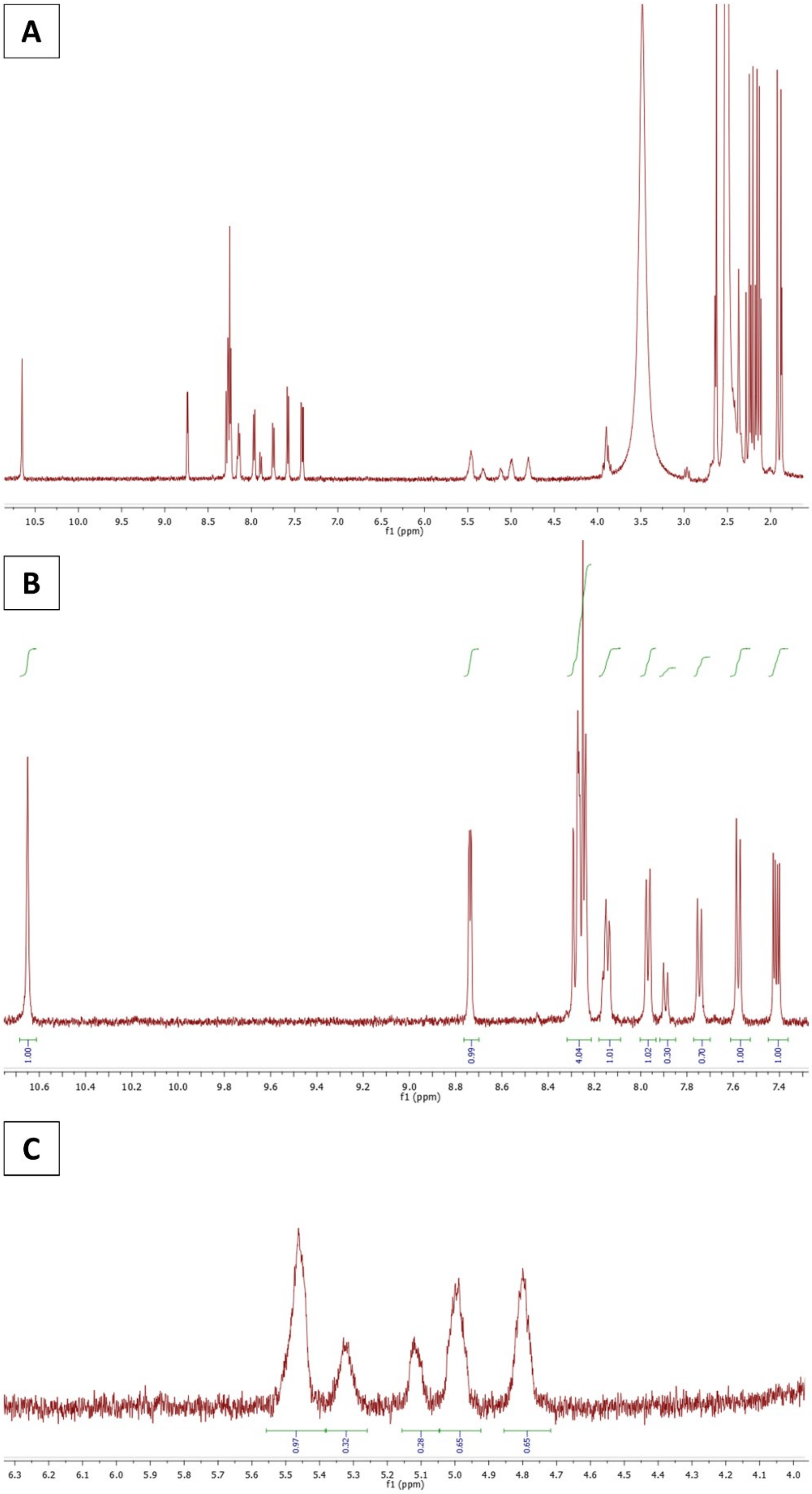

Figure S4: ^{1}H-NMR spectrum of **Co(Meacac)$_2$L$_{Pon}$,** showing (A) the whole-range, (B) the aromatic area from ~7–11 ppm and (C) the aliphatic area from ~4–6 ppm . Two sets of signals can be observed, but only in the aliphatic region and for the proton at 7.75/7.89 ppm.

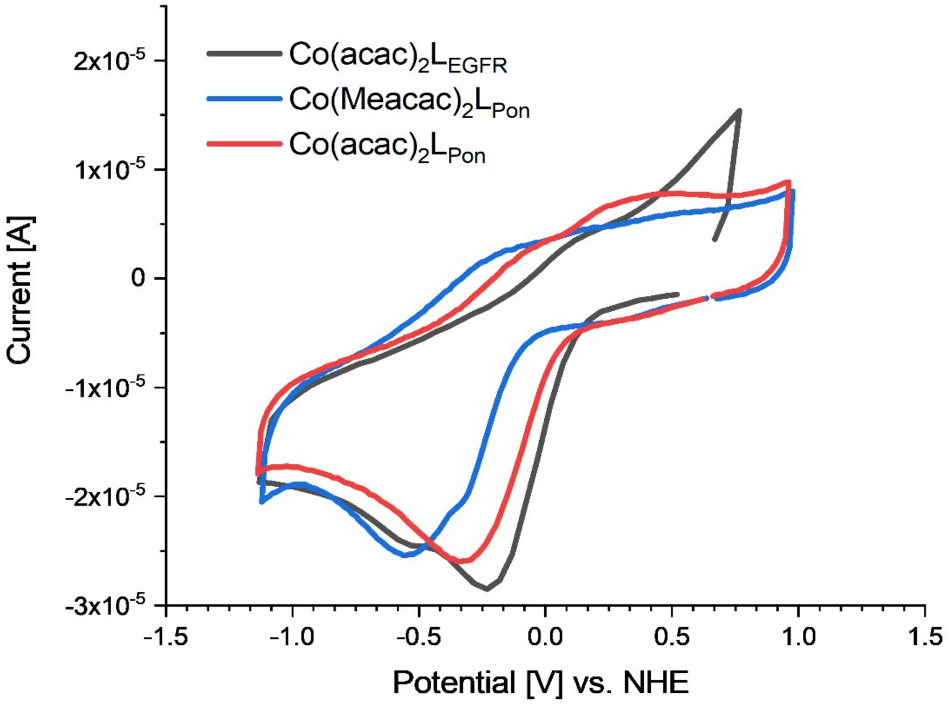

Figure S5: Cyclic voltammograms of **Co(acac)$_2$L$_{Pon}$, Co(Meacac)$_2$L$_{Pon}$** and **Co(acac)$_2$L$_{EGFR}$** in DMF (1.5 mM complex, I = 0.2 M [n-Bu$_4$N][BF$_4$], scan rate of 1000 mV/s, 25.0 °C).

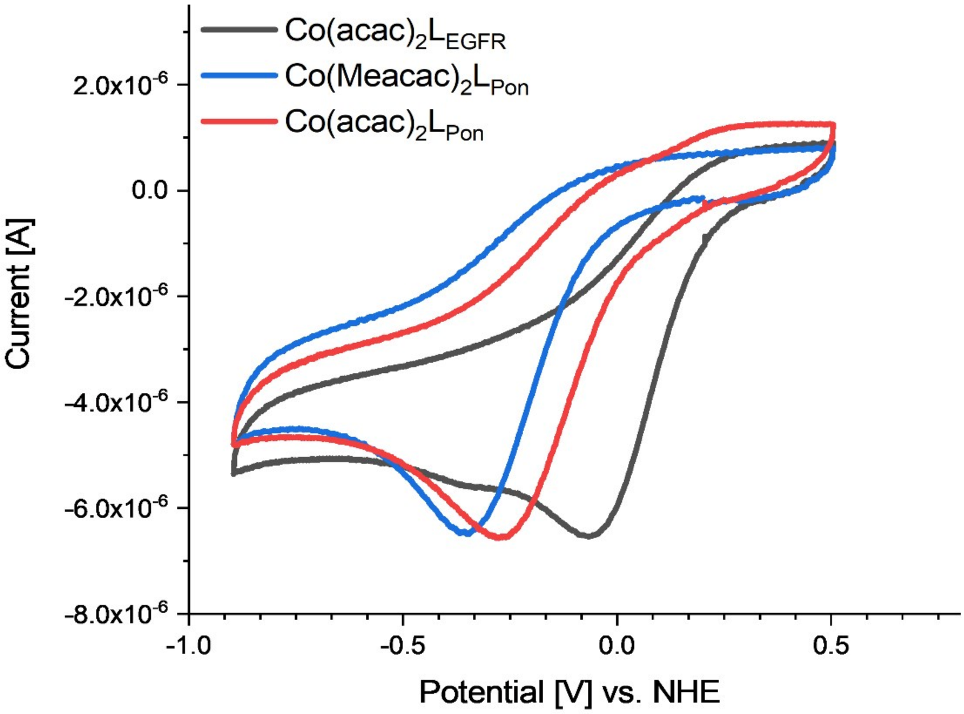

Figure S6: Cyclic voltammograms **Co(acac)$_2$L$_{Pon}$, Co(Meacac)$_2$L$_{Pon}$** and **Co(acac)$_2$L$_{EGFR}$** in 7:3 DMF/H_2O (1.5 mM complex, I = 0.2 M [n-Bu$_4$N][BF$_4$] for DMF, aqueous phase = 10 mM phosphate buffer at pH 7.40 with I = 0.1 M KCl, scan rate of 100 mV/s, 25.0 °C).

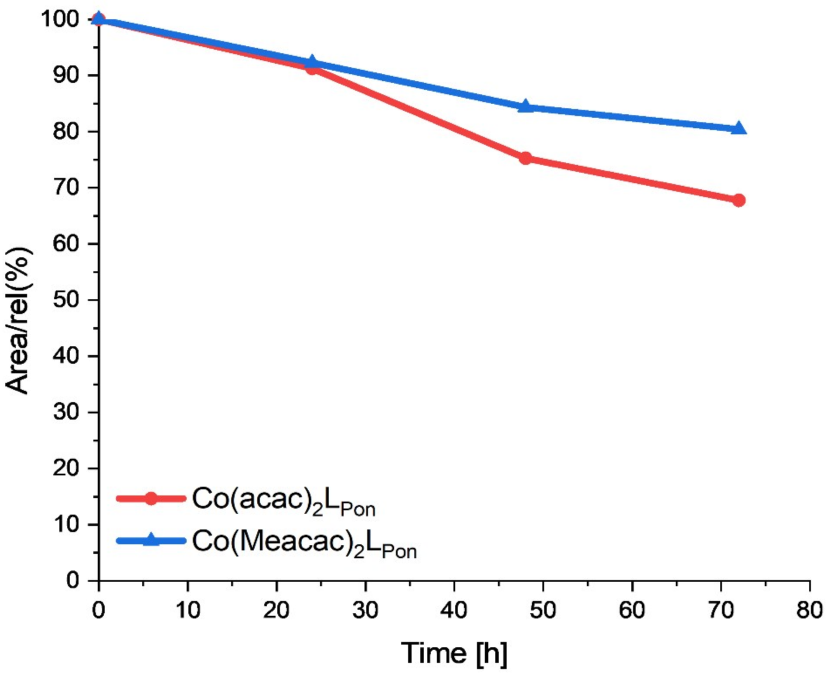

Figure S7: Stability measurements of **Co(acac)$_2$L$_{Pon}$** and **Co(Meacac)$_2$L$_{Pon}$** incubated in FCS at 37°C (pH 7.4, 150 mM phosphate buffer) analyzed by HPLC-MS over a time period of 72 h. The y-axis shows the relative ratio of the integrated peak areas of the intact complex over time (in percent) compared to the area at the starting point (0 h).

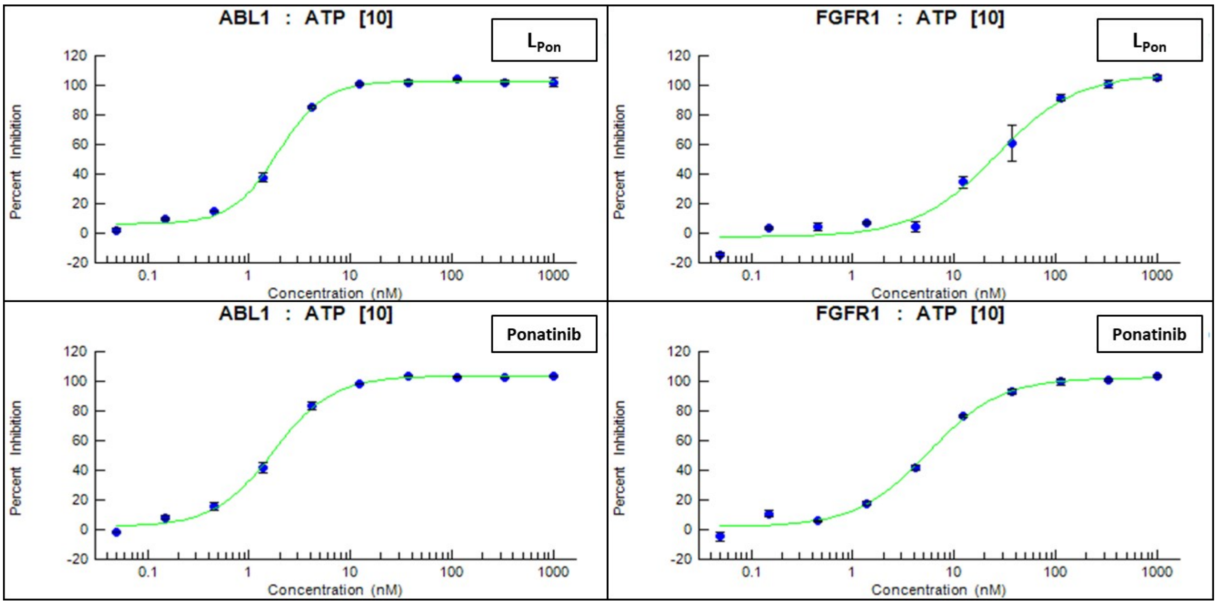

Figure S8: Data points of the cell-free ABL1 (left) and FGFR1 (right) kinase inhibition assay of **L$_{Pon}$** and ponatinib.

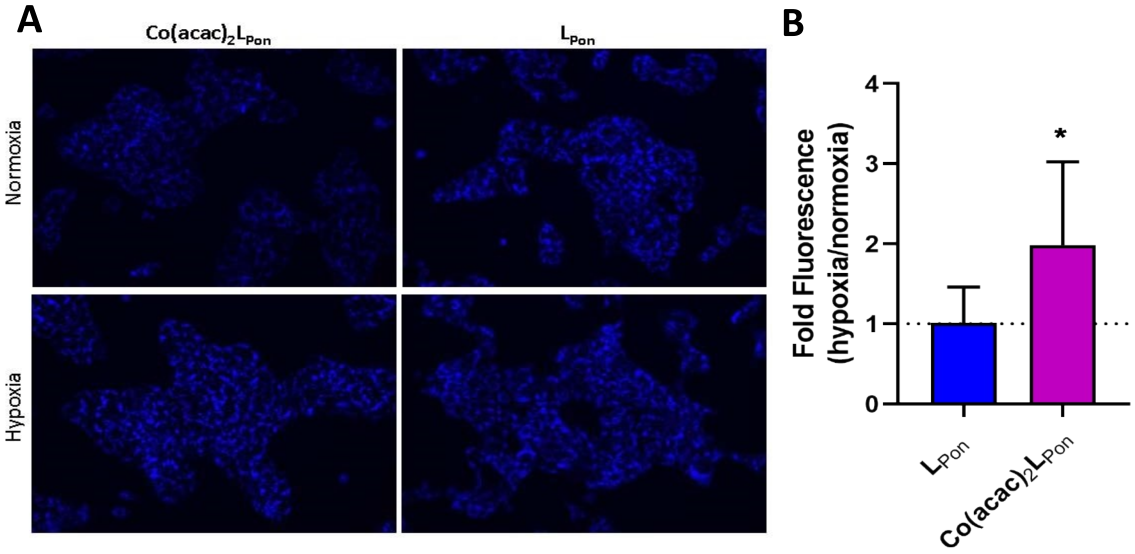

Figure S9: Ligand release of **Co(acac)$_2$L$_{Pon}$** under normoxic and hypoxic cell culture conditions in comparison to **L$_{Pon}$** using UV fluorescence microscopy. UM-UC-14 cells were incubated with 10 µM of compounds for 24 h. Images of the different treatments were taken by UV fluorescence microcopy (20X objective) and the corrected total cell fluorescence was evaluated using ImageJ software. Data in Figure S9B are given as means ± SD of ten analyzed microphotographs per compound. Statistical significance was calculated by unpaired t test with $p < .05$(*).

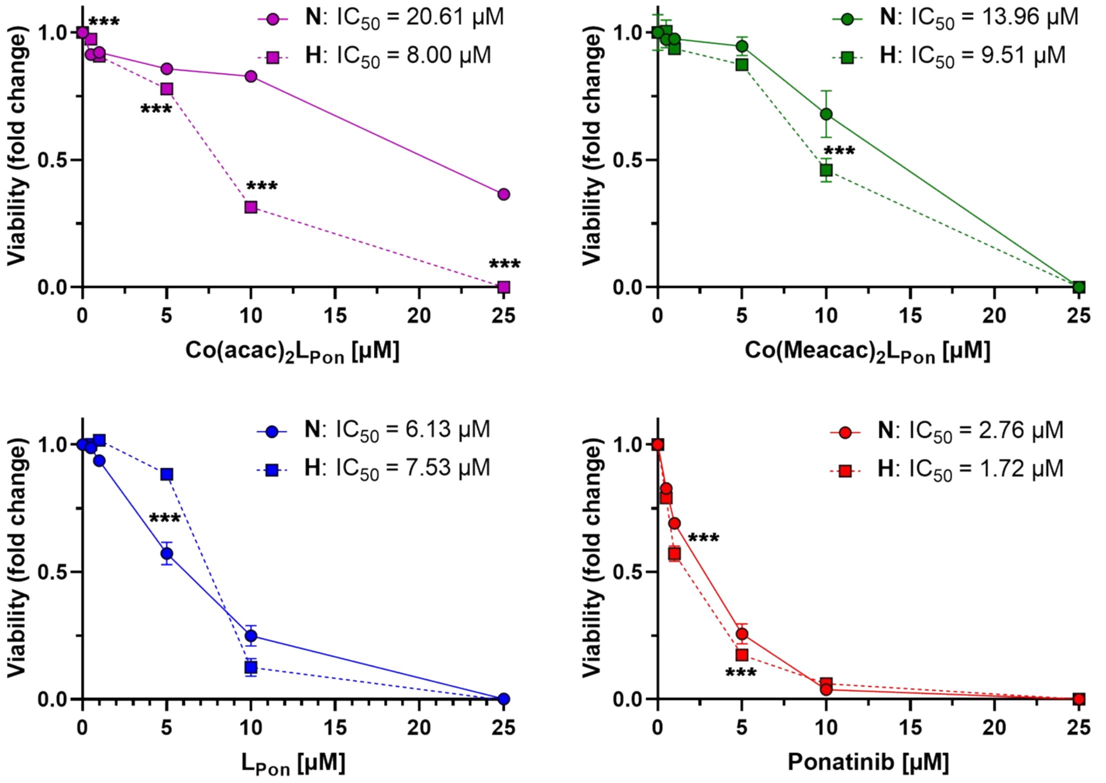

Figure S10: Cytotoxic activity of **Co(acac)$_2$L$_{Pon}$**, **Co(Meacac)$_2$L$_{Pon}$**, **L$_{Pon}$** and **ponatinib** against the UM-UC-14 cell line. Cells were incubated with the compounds under normoxic (21% O_2) or hypoxic conditions (0.1% O_2). ***N*** = normoxia; ***H*** = hypoxia. Cell viability was measured by MTT vitality assay after 72 h. Values are given as means ± SD of one representative experiment, performed in triplicates. Statistical significance was calculated by two-way ANOVA with Sidak multiple comparison test with p < .05(*); < .01 (**); < .001 (***).

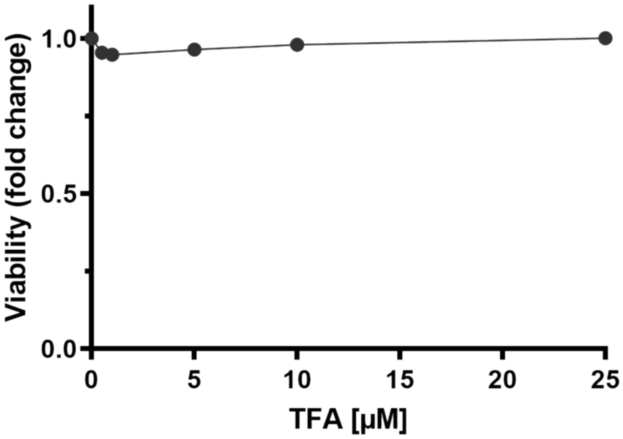

Figure S11: Cytotoxic activity of TFA against the UM-UC-14 cell line. Cells were incubated with TFA and cell viability was measured by MTT vitality assay after 72 h. Values are given as means ± SD of one representative experiment.

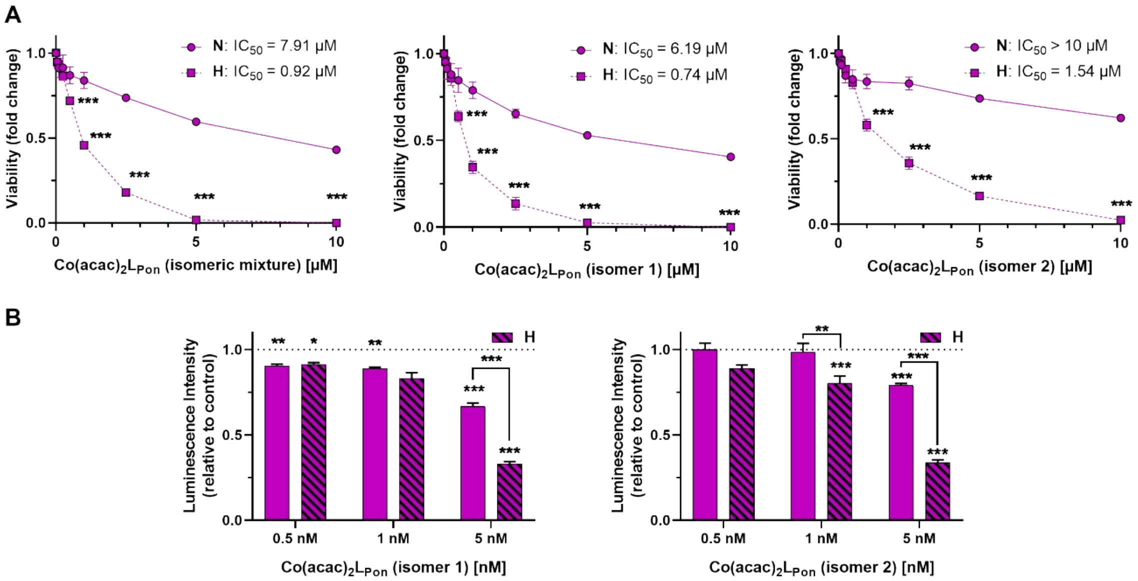

Figure S12: Cytotoxic activity of both pure isomers of **Co(acac)$_2$L$_{Pon}$** against human cancer cell models under normoxic (*N*) and hypoxic (*H*) conditions. A) FGFR3-driven UM-UC-14 urothelial cell viability was measured by MTT vitality assay and (B) cell viability of BCR-ABL-positive K-562 by luminescence assay based on ATP quantification (CellTiter-Glo) after 72 h. Data are given as means ± SD of one representative experiment performed in triplicates. Statistical significance was calculated by two-way ANOVA with Sidak multiple comparison test with p < .05(*); < .01 (**); < .001 (***).

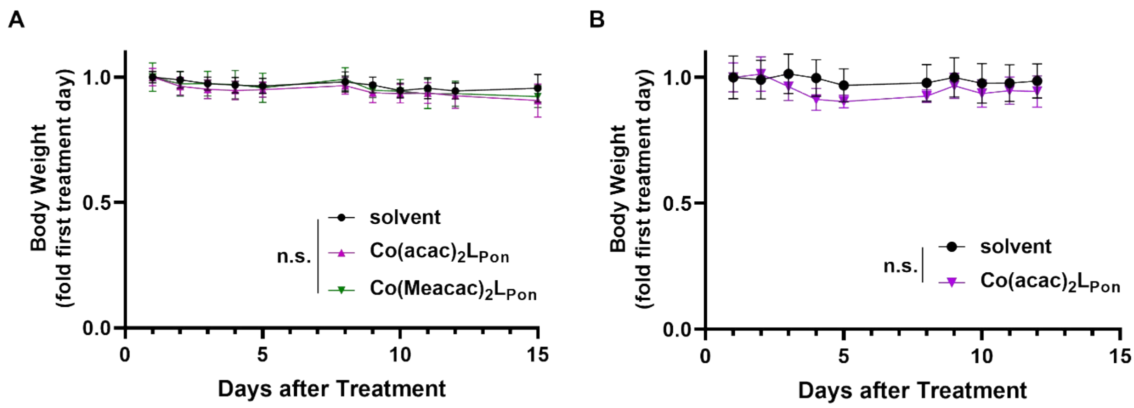

Figure S13: Animal weights of xenograft experiments. (A) BCR-ABL-driven leukemic K-562 cells or (B) FGFR3-driven urothelial UM-UC-14 cells were injected s.c. into the right flank of male CB17/SCID mice (n=4 animals per experimental group). When tumors were measurable (day 5 and day 7, respectively) compounds (10 mg kg^{-1} i.p.) were applied and animal weights were determined. Data are given as means ± SEM. Statistical significance was calculated by two-way ANOVA with Sidak multiple comparison test.
